# Moving the field forward: detection of epileptiform abnormalities on scalp electroencephalography using deep learning—clinical application perspectives

**DOI:** 10.1093/braincomms/fcac218

**Published:** 2022-08-29

**Authors:** Mubeen Janmohamed, Duong Nhu, Levin Kuhlmann, Amanda Gilligan, Chang Wei Tan, Piero Perucca, Terence J O’Brien, Patrick Kwan

**Affiliations:** Department of Neuroscience, Central Clinical School, Monash University, Melbourne, VIC 3004, Australia; Department of Neurology, Alfred Health, Melbourne, VIC 3004, Australia; Department of Neurology, The Royal Melbourne Hospital, Melbourne, VIC 3050, Australia; Department of Data Science and AI, Faculty of IT, Monash University, Clayton, VIC 3800, Australia; Department of Data Science and AI, Faculty of IT, Monash University, Clayton, VIC 3800, Australia; Neurosciences Clinical Institute, Epworth Healthcare Hospital, Melbourne, VIC 3121, Australia; Department of Data Science and AI, Faculty of IT, Monash University, Clayton, VIC 3800, Australia; Department of Neuroscience, Central Clinical School, Monash University, Melbourne, VIC 3004, Australia; Department of Neurology, Alfred Health, Melbourne, VIC 3004, Australia; Department of Medicine, Austin Health, The University of Melbourne, Melbourne, VIC 3084, Australia; Comprehensive Epilepsy Program, Department of Neurology, Austin Health, Melbourne, VIC 3084, Australia; Department of Neuroscience, Central Clinical School, Monash University, Melbourne, VIC 3004, Australia; Department of Neurology, Alfred Health, Melbourne, VIC 3004, Australia; Department of Neuroscience, Central Clinical School, Monash University, Melbourne, VIC 3004, Australia; Department of Neurology, Alfred Health, Melbourne, VIC 3004, Australia

**Keywords:** EEG, epileptiform abnormalities, automated detection, deep learning, epilepsy

## Abstract

The application of deep learning approaches for the detection of interictal epileptiform discharges is a nascent field, with most studies published in the past 5 years. Although many recent models have been published demonstrating promising results, deficiencies in descriptions of data sets, unstandardized methods, variation in performance evaluation and lack of demonstrable generalizability have made it difficult for these algorithms to be compared and progress to clinical validity. A few recent publications have provided a detailed breakdown of data sets and relevant performance metrics to exemplify the potential of deep learning in epileptiform discharge detection. This review provides an overview of the field and equips computer and data scientists with a synopsis of EEG data sets, background and epileptiform variation, model evaluation parameters and an awareness of the performance metrics of high impact and interest to the trained clinical and neuroscientist EEG end user. The gold standard and inter-rater disagreements in defining epileptiform abnormalities remain a challenge in the field, and a hierarchical proposal for epileptiform discharge labelling options is recommended. Standardized descriptions of data sets and reporting metrics are a priority. Source code-sharing and accessibility to public EEG data sets will increase the rigour, quality and progress in the field and allow validation and real-world clinical translation.

## Teaser

Janmohamed *et al.* report on clinical perspectives in the field of automated interictal epileptiform discharge detection. The article covers several considerations on the topic, including the variety of EEG sources and epileptiform data that may be encountered, inter-rater reliability in epileptiform labelling, lack of standardization in recent literature, metrics clarity and recommendations towards improving model reporting and clinical translation.

## Graphical Abstract

**Graphical abstract:**
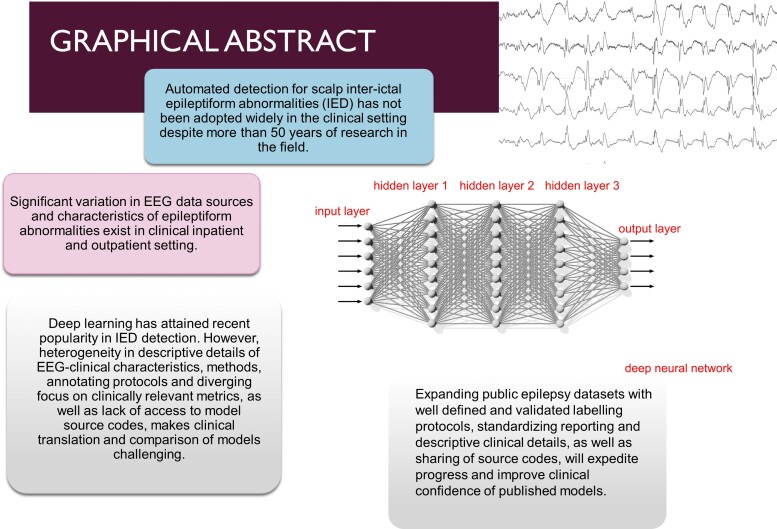

## Introduction

Research in computer-assisted automated detection of interictal epileptiform discharges (IEDs) transpired in the decades after EEG acquisition systems became available in clinical practice. The goal was to computerize detection of the ‘sharp-transient’ hallmark in epilepsy patients.^1,2^ An early study pursuing this goal was done in the early 1970s,^3^ where a now antiquated computer (PDP-12) was used to discriminate a waveform from a moving average derived from similar polarity amplitudes of 128 preceding waveforms. An indicator pulse was generated when the difference of a waveform amplitude reached a critical ratio. From that time onwards, modern research has explored quantitative time–frequency algorithms as well as machine learning (ML) strategies to develop mathematical models with the intent to achieve reliable automated IED detection.^4^ A range of methodologies have been employed to date often in combination, including template matching, autoregressive methods, mimetic analysis, power-spectral analysis (fast Fourier transform, Hilbert and Walsh transform), wavelet analysis, independent component analysis methods and neural network ML methods.^5^ However, these methods were tested only on small data sets,^6^ thus resulting in low generalizability. Larger data sets improve model performance but require time-consuming feature engineering process.^7^ Deep learning (DL), a relatively young field within ML, opens up a possibility to implement modern computing power to detect IEDs and improve workflow efficiency in EEG laboratories. DL differs from traditional ML by using multiple mathematical functions and has the advantage of automating latent feature extraction rather than manual feature selection, making the supervised aspect of training and learning from large data sets more efficient.^7^

A great deal of enthusiasm has been raised regarding DL outperforming expert specialists in healthcare diagnosis and clinical decision-making, and a considerable amount of diagnostic, prognostic and treatment-based ML experimentation has been pursued and published across medical subspecialties. These studies continue to make headlines in various fields. As an example in skin lesion detection, the classification of lesions into melanoma versus benign nevi has shown convolution neural networks (CNNs) outperforming dermatologists in dermoscopic examinations.^8^ In another landmark study for identifying and grading diabetic retinopathy using retinal fundus photographs, a DL neural network showed above expert-level sensitivity and specificity of over 90% in detecting referable diabetic retinopathy and macular oedema.^9^ This required labelled imaging by 54 ophthalmologists on a data set of 128 000 images for training and validating and testing in a subsequent data set where it outperformed health experts.

Such examples of remarkable success however should not be prematurely taken to conclude that ML in health has reached an implementational level in real-world clinical practice. When the above promising retinal classification model was deployed in a real-world prospective study in Thailand, several impediments were identified affecting system performance.^10^ Twenty-one per cent of the retinal photographs were rejected by the algorithm as they did not meet the system’s high standard for grading even when they were of adequate quality to be graded by the human visual eye. Real-world clinical data on the ground are frequently affected by a diverse range of technicalities which health experts have to regularly deal with, and this is particularly pertinent in the EEG and epilepsy world.

DL and ML in the EEG field covers a broad scope of research including epilepsy, sleep diagnostics and brain–computer interfacing.^11,12^ Within clinical epilepsy itself, ML approaches have been investigated for seizure detection,^13,14^ seizure prediction,^15^ epileptiform detection,^16^ epilepsy imaging, genetic mining and classification, medical^17^ and surgical treatment decision-making and clinical outcome prediction.^18^ High discriminative abilities have been asserted in these varied fields; however, there remains an uncertain perspective of real-world implementation and generalizability.

A recent study related to EEG IED detection employed a 10-fold cross-validation method on over 13 262 IED candidate waveforms.^19^ A very impressive area under the curve (AUC) of 0.98 of IED detection was cited for a DL model developed and termed as SpikeNet. Additionally, an AUC of 0.847 was also reported for classifying whole EEGs using a binary classifier trained using 10 extracted features. The model reportedly outperformed fellowship-trained EEG experts to detect individual IEDs. This number, however, needs to be contextualized. All data were obtained from a single centre including training and test data set and an external out-of-hospital test data set was not employed. An epoch-based graphical user interface point and click format (NeuroBrowser) was employed which required blinded classification by the raters. The overall inter-rater reliability in this particular study for these blinded reviewers agreeing on candidates as spikes was only fair with (Gwet κ) of 48.7. Most importantly, the source code for this model has not been available on public repositories to validate on external independent data sets. This external validation limitation in ML is well known.^18^

This review summarizes some of the perspectives of clinicians who have provided clinical support in DL IED detection in collaboration with data scientists via EEG data obtained from tertiary epilepsy centres in Melbourne. The article will allow data scientists and researchers entering the automated IED detection field to quickly understand the basic nature of the EEG data used in epilepsy management, challenges they will encounter upon embarking their journey and recommendations on moving the field forward.

### Search strategy and selection criteria

References for this review were identified through searches of PubMed with the search terms ‘inter-ictal, ‘epileptiform’, ‘spike’, ‘deep learning’, ‘automated software’ and ‘epilepsy’ from 2010 to January 2022. Only papers published in English were reviewed. The final reference list was generated on the basis of originality and relevance to the broad scope of this review.

### Why research in this field and limitations

The digital era has opened itself to automating tasks requiring human efforts, especially those which are repetitive and time-consuming (Table 1). This pursuit has been embarked to make hospital workflows more efficient. A routine EEG recording of 30 min usually takes anywhere between 5 min and an hour (median: 13 min) to be visually assessed and reported by an epilepsy specialist, depending on various factors, including presence of abnormalities, length of the EEG and artefacts present.^20^ This time can also be increased or decreased based on the setting of the EEG. In the intensive care unit (ICU) setting, 24 h of abnormal continuous EEG being reviewed for only seizure identification required a median of 44 (±20) min in a retrospective review of conventional review versus quantitative EEG comparator study.^21^ In contrast, a DL algorithm can take minutes to label and provide prediction labels for a 24 h EEG. A recent paper showed an average computational time of 7 s to label signal lengths of 1 h.^22^

**Table 1:** Pros and cons of future computer-assisted detection in EEG laboratories

Pros
Speed labelling and substantial data reduction leading to faster workflows Substituting unavailable expertise in low-resource countries Artificial intelligence is purported to have the potential of better results than traditionally trained experts.
Cons
Missed true epileptiform discharges (false negatives) with the potential to delay treatment Exaggerated labelling of artefacts as abnormalities (false positives) (see Fig. 3) Reduction of job and learning opportunities for EEG scientists and epilepsy trainees

During manual review, Identification and interpretation can take longer for more difficult and complex EEG data. An example would be intracranial data of a patient with a complex epileptogenic zone and several dozens to hundreds of electrode contacts resulting in a vast number of channels to review. Inconsistent labelling is also common in practice as different EEG technicians and clinicians use different approaches and terminology in marking data. A successful computer-assisted detection would theoretically vastly reduce the time and improve quality of labelling done manually by EEG scientists, technicians and clinicians.

The most concerning limitation of implementing automated detection in future workflows would be misreporting of an EEG by an overseeing clinician, in particular a non-expert epileptologist, biased by the automated programme. Every EEG has variation, and no model will ever result in a 100% accuracy. An EEG may be reported as positive when not, resulting in unnecessary and even harmful treatments being implemented, and conversely false negatives may delay treatment with the potential to cause harm to patients. This problem has also been often noted in global clinical practice, outside the expert neurophysiology community and addressed in a series of articles in 2013 appearing in Neurology. In a survey of 47 trained neurophysiologists, during the annual meeting of the ACNS in 2010, many noted coming across misread EEGs and 38% encountering them frequently.^23^ In a more recent study in India,^24^ 1862 EEGs were prospectively performed to identify the prevalence of benign epileptiform-like variants (BEVs). Under recognition and misreporting were common in the neurology community. Amongst 101 subjects whose previous raw EEGs were accessible, 30% of benign variants were noted to be misinterpreted as epileptiform abnormalities. Several recommendations and guidelines across the epileptology literature^23,25,26^ have been made to reduce this risk and efforts are in place to increase the training, teaching and reporting of EEGs. In the hands of inexperienced EEG readers, an automated detection programme may confound and potentially worsen misreporting.

### Overview of scalp EEG data sets available for automated detection

**Figure 1:**
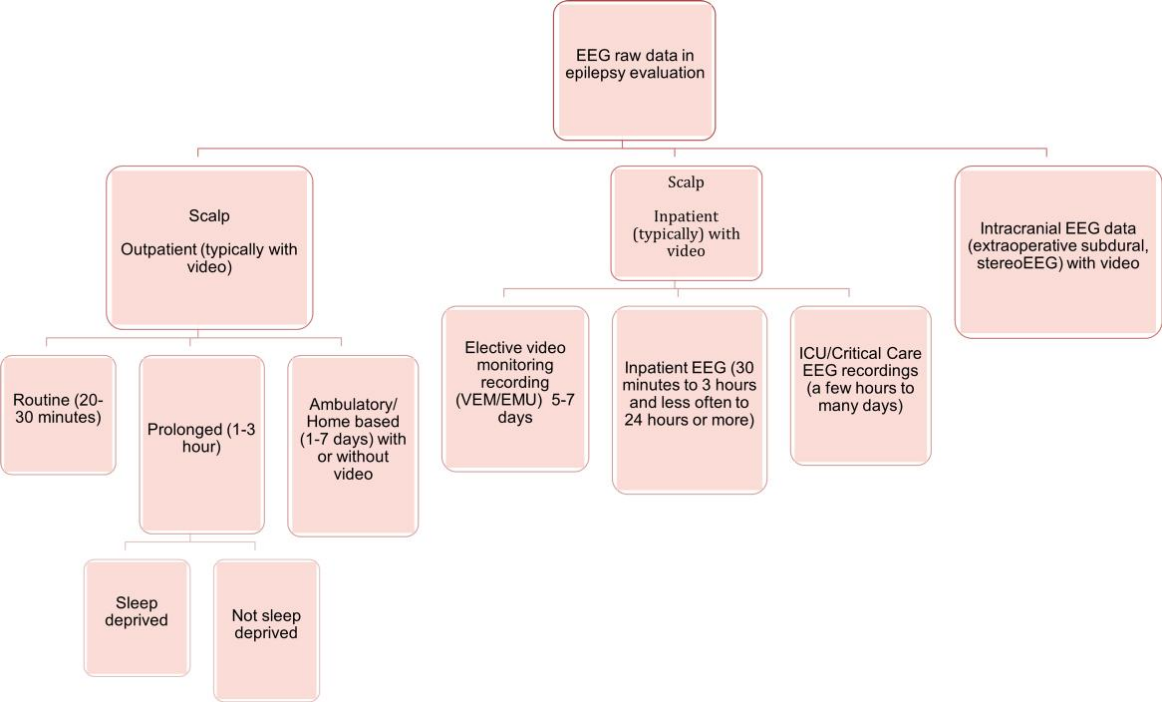
The structure of data available from hospital-based EEG servers.

A wide array of EEG recording types can be retrieved from hospital-based EEG servers (see Fig. 1). A scalp outpatient routine EEG is the simplest of the raw EEG data sets available and is usually recorded in a 10–20 electrode configuration, with or without ear electrodes. Routine EEG recordings are frequently done in rested patients who are not in an unwell clinical state and can generally, at most times, follow instructions. The quality of the EEG signals would be amenable for machine and DL as recording technicians in real-time are able to improve the quality of the signal recording and annotate important segments for a further clinician’s review. This has been a common data set used in DL literature. Routine outpatient EEGs typically range between 20 and 30 min and sometimes a more prolonged 1–3 h sleep-deprived or non-sleep-deprived EEG may be requested by the clinician overseeing the patient's care. Sleep-deprived EEG similarly provide good quality recordings given artefacts from movement and muscle are considerably reduced during sleep, and a marked surge in epileptiform abnormalities is seen in both focal and generalized epilepsy during sleep.^27–29^ Sleep, however, presents a different overall background from which the epileptiform abnormality emerges, and the epileptiform abnormality can present different morphologic characteristics and of briefer duration in the case of genetic generalized epilepsy.^30^

A downside of routine EEGs is that they are less likely to have abnormalities to gather for the training data set given their shorter duration. However, major tertiary referral centres however would still have several hundred to thousands of routine outpatient EEGs that are abnormal and contain epileptiform abnormalities stored on their servers depending on the protocol of archiving used and format compatibility with modern software. In a hospital, an EEG recording can also occur in a ward-based inpatient setting, a multiple-day elective video monitoring setting or the critical care setting. Video-EEG recordings of patients who are electively admitted for a multi-day recording would be intermediate in quality. Scientists are able to correct loose electrodes and aim to reduce artefact contamination, improve impedances and educate the inpatients to aim for better technical recordings. Sleep background is also available, and video is always available to correlate abnormalities for review. Here the number of electrodes can differ depending on the purpose of the elective admission. Recordings for surgical localization regularly have additional sub-temporal electrodes. Sometimes symmetric or asymmetric higher density electrode coverage in addition to the standard 10–20 electrode placement system may be carried out in different regions of the brain which introduce variation. Such video-EEG recordings can easily be processed to a 10–20 format for further data processing.

Lesser quality data sets would include ambulatory EEG^31,32^ recorded when patients are up and about at home, introducing large movement and myogenic artefacts and where an overseeing scientist is not reviewing the record until the leads are removed the next day or at the end of recording duration. Perhaps, most challenging of all would be critical care patients where electrical interference from surrounding equipment causes significant artefacts, electrodes may be placed in non-traditional positions or excluded due to craniotomies and the background may be confounded by sedative medications or the underlying brain insult. Prolonged ICU continuous EEG is often performed in hospitals for monitoring seizure activity of critically unwell patients.^33^

### The nature of EEG background and epileptiform discharges

**Figure 2:**
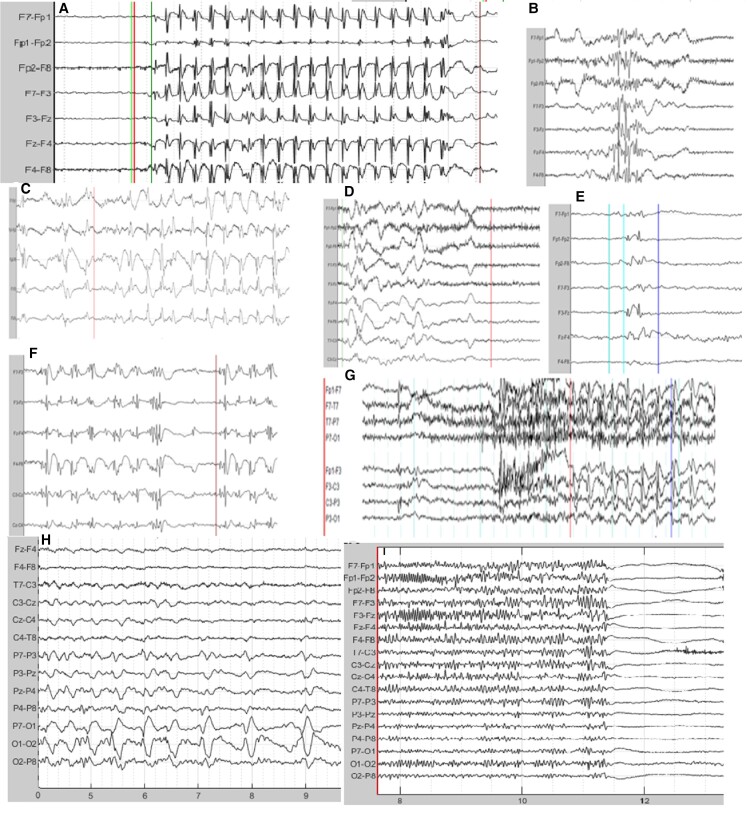
**Epileptiform variation in Genetic Generalized Epilepsy EEG data sets.** (From left to right) (**A**) Classic 3 Hz spike and wave on transverse montage, (**B**) polyspikes with EMG artefact in frontopolar channels and eye movements, (**C**) slow spike/wave on transverse montage, (**D**) mild EMG affecting frontal channels with embedded small spike and waves and irregular slow waves, (**E**) fragments on transverse montage, (**F**) polyspike/slow waves on transverse montage, (**G**) marked EMG artefact confounding epileptiform abnormality in temporal and frontal channels on longitudinal montage, (**H**) a train of focal posterior sharp waves and a (**I**) generalized paroxysmal fast burst.

The EEG presents a wide diversity and dynamic nature of both background and epileptiform discharges in an EEG. This variation has to be understood before embarking on the ambition of a universal IED detection model. Figure 2 demonstrates sample EEG epochs of epileptiform variation in a genetic generalized epilepsy data set showing a sample of diversity in epilepsy IEDs for one epilepsy type.

**Figure 3:**
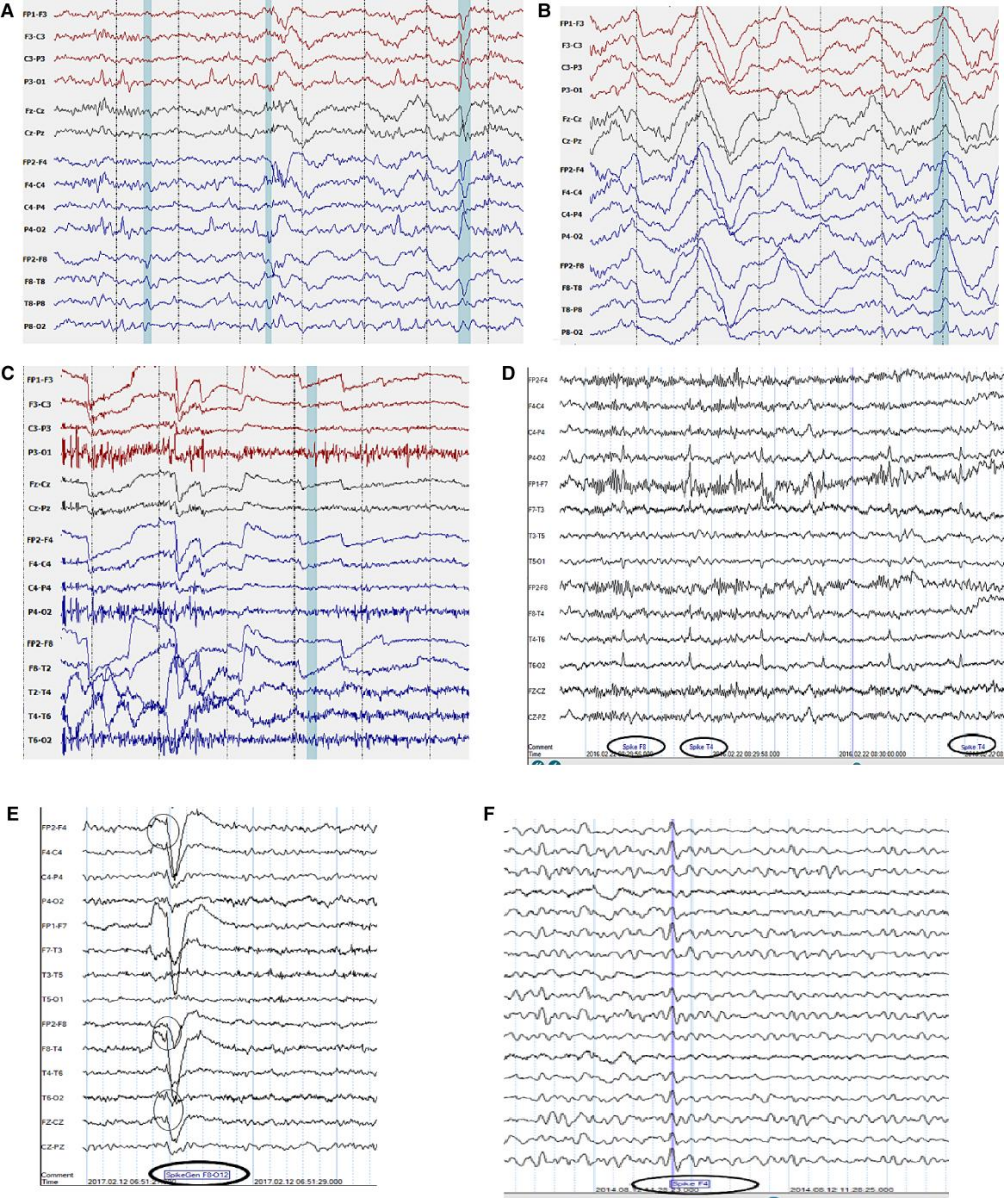
**Artefacts mimicking interictal epileptiform abnormalities.** IED mimics (**A**) *v*-wave mimicking sharp wave and is labelled as abnormal by algorithm. (**B**) High-amplitude slow wave in Stage 3 sleep causing false positive, (**C**) ocular artefact, (**D**) ECG artefact picked up as runs of IEDs, (**E**) lateral rectus spikes and (**F**) wicket spike picked as false positive.

The normal background of an EEG is dynamic and can be divided into normal awake, drowsy and sleep stages.^34^ Background frequencies are slower and less dynamic in encephalopathic patients or those with developmental delay and neurodegenerative conditions. There can even be an association of background frequencies with age. Paediatric EEG has a much more complex range of normal background while older patients demonstrate slowing of the dominant posterior alpha activity. Simple state-occurrences such as eye closure can also modulate the background. A wide multitude of technical issues and artefacts can add to tremendous variation in the background during the awake state and some of these can resemble epileptiform abnormalities.^35^ These can occur in both the awake and drowsy state. Several background variants of the normal EEG can occur including alpha variants (fast and slow) and posterior slow waves of youth. Further, BEVs can occur in the EEG during awake and drowsy state and may include Benign small sharp spikes (or Benign Epileptiform Transients of sleep, BETs), wicket waves, 14 and 6 positive spikes, and 6 Hz phantom spike and wave.^34,36,37^ Physiologic changes in the drowsy and sleep record include attenuated posterior rhythm, central theta, V-sharp (vertex) waves, large K-complexes, spindles, arousal patterns, positive occipital sharp transients, as well as temporal or diffuse rhythmic theta and high-amplitude delta slowing.^38^ Detection models may confuse some of these morphologies with epileptiform discharges (see Fig. 3), especially those discharges showing rhythmic sharply contoured waves forms or even delta duration slow waves, such as the kind seen in spike/slow wave.^39^ Similar to dynamic changes in background which characterize a normal or abnormal EEG, there is no uniformity in epileptiform discharges within and across patients. Epileptiform discharges can vary in duration, morphology, periodicity, topography (Fig. 2) and can be modulated by either state changes or other provocative manoeuvres. The diversity of epileptiform discharges can include spikes (20–80 ms), sharp waves (80–200 ms), spike–slow wave, sharp-slow wave, polyspikes, polyspike–slow waves, as well as fast activity associated with the aforementioned. These can be located, within one subject, in one region or in two or more regions either in one hemisphere or bilaterally. When these engage bilateral networks, they are referred to as generalized epileptiform abnormalities and when confined to one hemisphere in a few electrode sensors as focal abnormalities. They can occur as isolated transients or be part of a sequential train or run which can be periodic^40^ or quasi-periodic. In some patients, these frequently recur through the duration of an EEG recording but may only occur only occasionally in briefer recordings. They can occur in combination with any of the background states mentioned above.

Sleep modulates and accentuates the occurrence of epileptiform discharges^30^ in focal, as well as in generalized epilepsy and so does provocative manoeuvres which in different epilepsies may include photic stimulation,^41^ hyperventilation or even things such as visual, audio or cognitive tasks.^42,43^ An ambitious all-spike (universal IED detecting) DL model should have undergone training with a vast amount of EEG capturing the majority of this variation. The differences further are confounded by EEGs available from different settings, where different noise levels will be present. To reiterate, an outpatient ambulatory EEG recording is not equivalent to a routine resting EEG in terms of background quality and both of those will be different to EEGs acquired in a critical care EEG.

### Inter-rater agreements and the gold standard comparator

One of the main concerns in current DL and ML studies is the lack of an unambiguous framework of what the gold standard or ‘ground-truth’ is for determining the accuracy of the final computing model.^44^ Given the consistent real-world underperformance in the reliability of computers versus humans in EEG IED discretion, the reference standard today remains visual review and classification by a trained epileptologist or clinical neurophysiologist. This presents some limitations as inter-rater reliability amongst EEG readers has been controversial due to the perceptual phenomenon and probabilistic art of reading spikes.^45^ For decades, the question of inter-rater agreement (IRA) has been investigated in parallel with the question of computerized detection. The Food and Drug Federal Administration requires three electroencephalographers (EEGers) in the annotation process for approval of an algorithm.^46^ Some authors have directly looked at inter-rater reliability and specify the number of EEG raters selected for an internal criteria of a definite IED.^19^ Halford^5^ mentions at least four different agreement criteria used by ML authors for referencing actual IEDs in his review paper including concordance between all raters, a cut-off number of raters from the whole group, reconciled rating amongst reviewers and some papers using only one rater to define the gold standard. Expert pooling has been found to be better and larger group sizes from 3 to 10 have been reported to be ideal yet judiciously selecting expert EEGers for IED annotation research projects may reduce the need for this number.^46^

Although the above may lead to a perception that IRA amongst EEGers is imperfect and unreliable, this is not entirely accurate. Several studies have shown moderate to substantial IRAs with some studies report higher Gwet or kappa as well as high performance of blinded clinical experts compared to an unblinded gold standard.^47–49^ IRA in ‘whole EEG’ categorization remains high given low-perception spikes are contextualized by EEGers before concluding the report as normal and abnormal. A limitation of poor or fair IED agreement studies is that reviewers are blinded to clinical context or deal with very short segments of data when tested.^50^ Most real-word EEG review of waveforms requires an awareness of patients’ age, compliance with recording instructions, technical quality, sedative agents, pharmacologic agents, previous EEG characteristic, clinical context and the conscious state of the patient during recording.

A recent paper provides a good benchmark and sheds light on what can be used as a gold standard. Kural *et al.*^47^ assessed six criteria that are used to determine what is or is not an epileptiform abnormality to assess inter-rater variability amongst clinicians for each feature criteria and assess the International Federation of Clinical Neurophysiology (IFCN) criteria as a whole for validity. In the study, they used a strict methodology to confirm an IED. It required two reviewers agreeing that the candidate waveform was a sharp-transient and furthermore additional criteria of a patient not only to have confirmed epilepsy but of the selected transient being concordant with the patient’s recorded seizure and location as expected in that syndrome or focality (interictal, ictal and syndrome correlation). The clinical context was thus extensively incorporated in the decision-making of what is or is not a spike thus setting an acceptable benchmark for gold standard for the purposes of that study. With that gold standard other methods of defining epileptiform were evaluated. Blinded reviewers utilizing four or five of the six IFCN criteria together provided a strong accuracy of the waveform being labelled as epileptiform with accuracy levels of 91% (95% CI: 83.6–95.80) and 88% (95% CI: 80–93.6) against the gold standard. Furthermore, experts solely using their clinical experience with no protocol method and simply consensus provided a 92% (95% CI: 84.8–96.5) accuracy. It is important to note that all these experts were blinded to the original two reviewer unblinded gold standard assessment. The six IFCN criteria used included the morphology of spikiness or sharpness, asymmetry of ascent–descent slope, duration difference from background, an after-going slow wave, background disruption and a concordant voltage map.

### Assessment of current DL studies

A systematic review by the authors submitted and under review examined 17 recent DL scalp EEG studies for IED detection (Supplementary Table 1). Of the studies, 60% used focal and generalized epilepsy EEGs, whereas the remaining focused on either or a BECT data set. Six studies used data from more than one centre for testing set. Two papers used more than a thousand EEG recording data set with median number of EEG recordings per study as *n* = 166. Routine EEG recordings were most commonly employed whereas prolonged recordings next most common. All studies utilized supervised or semi-supervised learning requiring labelled data; however, significant variation in gold standard identification of IEDs existed. Montage applications were mentioned in some studies and not mentioned in others with the most common channel combination for data input as longitudinal bipolar montage. The most common architecture employed for DL was CNN and long short-term memory with combinations and hybrid comprising the rest. Accuracy-based measures AUC/balanced accuracy or simply accuracy were reported in all studies; however, sensitivity in combination with either false positives or precision was not reported in some studies. Overall, a wide variety of data preparation, pre-processing methods and neural architecture techniques were utilized. Presentation focus of performance metrics vary significantly amongst studies introducing difficulty in comparisons of strength of models. Further technical details of the data properties, pre-processing methods, design of DL architectures and layers, optimizers and pooling methods can be reviewed from the original papers and a systematic review by the authors is under review.

### Annotation standards

Recent literature on DL IED detection do not usually elaborate on the clinical annotation protocol used for the supervised learning process and we found this information lacking in publications. A six-way labelling classification of epilepsy EEGs is employed by the Temple University group which has availed their public data set on the internet.^51^ Proper annotation labelling may be important for the performance of the algorithm. An abnormality can be marked using a single marker or a start and end marker. The windowing method employed for training and testing purposes for IED detection may incorporate partial normal segments in the windows designated as abnormal containing epileptiform abnormalities. If only one marker is used it can be placed at various points along the discharge most frequently at the negative peak of a selected spike portion. No strict rigour can be employed here due to the vast heterogeneity of how transients and prolonged discharges appear. Even when a more laborious ‘start’ and ‘end’ markers are utilized there is frequent inter and intra-rater variation from our centre’s experience in labelling as abnormalities often do not have clean onsets and offsets. A decision may be made to annotate the first spike onset, however spikes may terminate before the discharge has ended in the case of generalized epileptiform abnormalities.^52^ Conversely, discharges may emerge with some abnormality in background or rhythmic slowing before showing clear spike morphology. In the case of stereotyped repeated focal transients, the onset and offset may be easier to define. An annotation marker may be generic with an instant timestamp without regard to the channels involved, or it may be specific and labelled according to the specific channels involved. The Temple University Hospital (TUH) events public corpus has made an effort to label abnormalities based on specific channels involved and may allow more precise abnormal signal input for the subsequent learning process.^53^ These annotations of the data sets however will need to be systematically validated.

### Metrics evaluation

Utilizing sensitivity, specificity and AUC of each model does not always translate into useful clinical assessment. There remains a challenge with reporting metrics in regard to IED DL models, and the most clinically useful metrics are variedly reported in the currently existing literature. Both accuracy and to some extent AUC as understood by data scientists in the field, unless contextualized with other metrics, can be misinterpreted by clinicians. This has been noted by some authors.^54^ Summaries of recent DL models consistently report over >0.9 or 90% AUC^19,55–59^ A model can be stated as having 90% ‘AUC’ or accuracy and yet be unreliable from the clinical perspective. This will occur if the normal windows for the majority part were correctly predicted even if the abnormal discharges, which occupy very brief lengths and occur sparsely, were all mostly missed. The only tuning of the model to give a high accuracy would be to reduce the number of false positives which could be attained by raising the detection or perception threshold (low sensitivity setting). True negatives in such a model will be high in both the numerator and denominator, falsely giving an ‘accurate performance’. An AUC does not always solve this problem as both a high sensitivity and specificity do not necessarily address the issue of false positives (examples in Fig. 3). This problem of imbalanced data set during ML and DL training occurs when normal background input vastly exceeds abnormal windows with a ratio of up to 1:1000.^56^ Data augmentation methods have at times been incorporated to increase the representation of the spike minority class using oversampling techniques.^60,61^

When evaluating a DL model, several other metrics, therefore, have to be taken into account and few metrics are helpful as standalone measures to give a perspective on success (see Table 2). Precision reports true positive IEDs in the entire set of predicted IEDs. It represents the positive predictive value in clinical terms. A higher precision implies a lower false positive rate. False positives per minute or hour is a simple and informative metric which provides accurate insight into performance when full-length EEGs are evaluated in the test data set. Precision or false positive rate coupled with sensitivity represent a better measure than specificity and AUC. Class imbalance as described above skews specificity TN/(TN + FP) and other measures dependent on it like AUC. Amongst other parameters of high utility is the F1-score which provides a weighted average of both sensitivity and precision and the AUPRC which is the area under the precision–recall curve (AUPRC). The F1-score weights the two most important variables and will take into account false positives and false negatives without contaminating or exaggerating performance with the imbalanced true negatives.

**Table 2:** Performance metrics commonly used in deep and machine learning studies

Metrics of clinical utility for IED detection
Sensitivity: Proportion of true gold standard IEDs correctly detected
Precision: The proportion of true marked gold standard IEDs to all machine predicted positive labels. (True positives)/(true positives + false positives)
False positive rate: Rate of false positives which were not classified by the gold standard as IEDs typically reported in per hour
F1-score—This takes into account the two most relevant metrics of precision and recall.
AUPRC—Area under the precision–recall curve (AUPRC) which differs from the area under the ROC curve. A model achieves perfect score when it identifies all epileptiform abnormalities without marking normal or benign abnormalities
Metrics of limited clinical utility in isolation
True negatives, specificity, accuracy and AUROC (area under ROC curve)

Channel-based labelling has its advantage. For focal abnormalities and artefacts maximal involvement is in few channels can improving the target specificity for data training. On the other hand, if one maximally involved channel is selected for training this has the risk of ignoring data from the remaining field extent of the IEDs. If per channel evaluation is desired modifications to appraisal will be required in a mixed unselected data set as generalized epileptiform abnormalities or spikes with broad fields will show preferential biasing compared with localized spikes. From the clinicians’ perspective, single-channel annotation would be tedious to review, present redundant data and pose difficulty in evaluation when many channels are involved. A single timestamp even for generalized or broad field focal abnormalities would serve the intended purpose of automated detection as a screening tool.

We found a lack of clarity in many papers as to how redundant predictions for a single contiguous discharge are dealt with. Continuous EEG data are frequently segmented into short windows (ranging from 0.5 to 2 s) and is trained or tested using a sliding window of overlapping or non-overlapping windows (50–75%). From a clinician’s perspective, redundant (repeated) markings between pre-labelled start and end marking of a contiguous epileptiform abnormality should not be counted as true or false positives and should not influence metric calculations. This may differ from the understanding of the detection target in which IEDs are thought exclusively as ‘spikes’. We reiterate in this article the many different types of epileptiform patterns including bi- or triphasic sharps, spikes, polyspikes, fast patterns with or without slow waves occurring in isolation or prolonged repetitive bursts exceeding conventional window segmentation (see Fig. 2) used in DL are common.

In the case of highly active prolonged abnormal EEGs, the recording can be trimmed to reduce margin of error for the manual annotator instead of using dozens of hours. Furthermore, manual review and validation of the automated spike labels should also be performed to ensure any extras detected by the ML are false positives and not detected but unlabelled true positive IEDs.

### Whole EEG classification

In this area of interest, some authors have reported on IED-free versus not-free to categorize whole EEG classification.^19,57,59^ This may implement a IED rate threshold to categorize EEGs into normal versus abnormal which differs from criteria used in real-world classification by epileptologist. Clinically, one unequivocal epileptiform abnormality suffices to classify an EEG as abnormal. If EEGs were sorted from clinical reports, caution needs to be adopted given other EEG features, most notably focal slowing or indeterminate findings, can lead to the classification of an EEG as abnormal without an epileptiform abnormality. Textual mining of reports for data set retrieval should specifically require the presence of epileptiform abnormalities and the conclusion of the report should be ascertained. Sensitivity and specificity calculations are easier to calculate for whole EEG classification as actual positives and actual negatives are easier to define without the challenge of defining windows in relation to IED durations. IRA on whole EEG classification is higher than for individual IEDs.

### Future directions and overcoming the IED challenge

In seizure detection, low false alarm rates (<1/h) and high detection rates (70–80%) have been achieved,^62^ and software are operational in some hospitals for seizure alerting, detection and continuous monitoring.^63^ On the other hand, despite more than 50 years of study in this study area, commercial or open-source software have not become pervasive in clinical use for the detection of IEDs on EEG recordings despite the immense benefit to time and labour challenges in an EEG laboratory. A recent commercialized spike detection software trained using DL algorithm, Encevis Solutions^64^ (Austria), sensibly uses clustering to overcome the high rate of false detections 112/h for a high sensitivity of 89%.^54^ Persyst 13 has been reported in one study as non-inferior in performance to senior EEG technologists^65^ at a low-perception predictive setting (high sensitivity setting) but was found to have much higher false positive rates at various perception thresholds compared with board-certified EEGers reviewing Epoch-based transients.^66^ It remains to be seen how well the software will perform on more varied, larger and longer unselected EEG data sets using a reliable gold standard. There remains significant scepticism amongst EEG technicians and clinicians as to the benefits of available software accurately guiding the process of capturing and labelling spikes on scalp EEGs and subsequently quantifying IEDs let alone precisely making a judgement on classification of a scalp EEG into normal or abnormal (IED-free versus IED-EEGs).

The scepticism and difficulty in readily available DL algorithms for computer-assisted clinical EEG reporting has been due to a great number of barriers. The wide heterogeneity of methods, statistical approaches and reporting in current literature introduces difficulty in comparison of models. The models may appear accurate in a single-centre data set but their applicability to multiple data sets is more challenging. Standardized descriptions of data sets and reporting metrics is essential remains a priority in this field.

### Proposed hierarchy for gold standard epileptiform detection

Given no consensus exists on the gold standard to be used for identifying and labelling epileptiform abnormalities for training and testing DL models, different approaches can be utilized based on extent of clinical support available in the centre. In the most reliable situation, epilepsy experts (even a few) assess each spike or sharp wave in relevant time epochs as an epileptiform abnormality having awareness of the context of the patient’s profile (clinical history and imaging) and have access to longer EEG recording or at least more than just short epochs, including ictal patterns and locations to correlate for concordance. EEG reviewing and reporting, especially for inpatient EEGs, eventually considers all clinical comments regarding why the EEG is performed. This criterion may not be practical for big data research projects. Second, despite the above-mentioned limitation, few sufficiently trained EEGers utilizing the four or five of the six IFCN criteria mentioned above, preferably with basic context for the EEG being labelled, would be a validated method.^47^ The third method would be epilepsy experts either marking via ‘experience’ without adequate exposure of the entire EEG and clinical context of the respective EEGs. Due to sub-par inter-rater concordance frequently cited in the literature, the third method remains controversial in terms of how many people should agree. Occasional papers, however, continue to show an adequate level of expert agreement, even when this method is employed.^47,48,67^

If there are limitations in obtaining epilepsy experts to annotate and determine ground truths, a layered approach can be implemented where a less rigorous method is employed to annotate the training data set and a more rigorous method used to annotate the testing data set. This will ensure that the performance results provided in the study have been compared against an adequate gold standard in that centre.

### Standardizing reporting of methods and results

A recent exemplary paper provides details to properly understand a DL publication of IED detection. Adequate description and division of EEG data was provided, epilepsy syndrome details, method and algorithmic details and most importantly comprehensive performance metrics results.^22^ For the data set, there should be a clear explanation of the recordings being either scalp or intracranial, the environmental setting in which the EEG was performed, and the breakdown of the type of EEGs used for both training and testing data sets. Additional clinical characteristics of cohort can be helpful. Extended labels are preferred to single timestamps during manual labelling otherwise the IED discharge will be assumed to be the window size by the performance evaluator to enable calculation of relevant metrics. If combinations of heterogenous recorded EEG data in different inpatient and outpatient settings are used, the proportion of different respective EEG types implemented in both the training and testing data set should also be described. Electrode configurations used and channel derivation from electrode montaging should be mentioned as these can introduce some differences in signal characteristics. Average referenced signals can be different depending on vertex or ear electrode referencing or all-electrode averaging which can be different from bipolar or transverse derived signals. The montage ultimately chosen for testing and training may introduce some variation in the derivative signal and may not translate well into a test data set using a different montage configuration for signal derivation. As an example, waveforms may appear sharp and phase reversing on bipolar whilst not appearing different from the background in an average montage. Similarly averaging can sometimes bring out waveforms which undergo differential voltage cancellation in bipolar montage due to equal strength amplitudes. In a recent study, we found combined training on transverse and longitudinal montages simultaneously provided high F1-score in a recent GCN convolution model.^39^

Very few publications provide details on epilepsy type, syndromes and nature of discharges and whether the bulk of the abnormal discharges or the majority background used was derived from awake or sleep state or in what estimated proportion. This can be important as a data set used to train a focal epilepsy model may not be appropriate for a generalized epilepsy test data set. Furthermore, some models may work well on awake background but present false positives in sleep EEG due to low frequency waveforms being confused with slow wave abnormalities.

Evaluation results are frequently present on test data sets in a summarized pooled manner. A few outlier EEGs causing poor performance may markedly skew the results to show the algorithm as inaccurate whereas this may be the case because of only a few EEGs in which the algorithm failed significantly. In our ongoing work evaluating an unpublished data set^39^ implementing a Graph convolution method (viewing an EEG montage as a graph theory using electrodes as nodes and pair linkage as edges), we found removal of 4 outlier EEGs from a test data set of 28 EEGs markedly improved precision at a 0.80 detection threshold from 28 to 63% with only a 10% drop in sensitivity (errant spike windows predicted reduced from 781 to 57). Outlier identification efforts, although time-consuming, should therefore be made in conjunction with a trained epileptologist to find the reasons why the overall results of an algorithm may be poor.

Standardized metrics should be reported including as many performance metrics as possible to provide a holistic view rather than a focus on accuracy or AUC. Most importantly and invariably clinical useful metrics of false positive rates and sensitivity should be reported within abstracts and conclusions. Other details on how the analysis and performance statistics were calculated should be elaborated in forthcoming DL studies. Were brief epochs or entire lengths of EEGs evaluated in the test data set to decide on prediction accuracy? Accuracy/AUC calculated on IEDs in a substantially imbalanced real-world data set of whole EEG recordings is different from Accuracy/AUC calculated on a data set limited to segmental review or epochs with an attempt to balance normal and abnormal segments in the test set. Selection bias can also be introduced into the epoch-based methods as noise-free segments with better technical quality, uncontroversial epileptiform discharges and more normative backgrounds with less complexity can be chosen by the data set retrieving team. The problem of imbalanced data set has to be tackled in this field as epilepsy data will always have the vast majority >95% or more of its signal to be normal background apart from a few outlier intractable epilepsy patients who have frequent, near continuous or continuous epileptiform abnormalities interspersing background. The benchmarking test data set must therefore be an imbalanced data set if any real-world clinical utility is to be desired.

### Public data sets and source code-sharing

Cross-testing is vital and will reveal the actual performance of a model. This has only started to be employed in seizure detection. False detections of seizures ranged from 0.15 per hour to 2.5 per hour depending on different data sets used.^68^ The hospital and locality ethics of sharing EEG data makes it complex for potential collaborators seeking to implement their model algorithms on external data sets. Multi-centre data set collation should nevertheless continue to be pursued. Epilepsy centres collaborating will be able to reach target numbers reached in imaging classification by share loading contributions and be able to allow the DL model to be trained on a large amount of morphologic, topographic and artefactual variation of windows containing epileptiform discharges. This cannot be done without a collaborative mindset.

Source codes detailing current DL models being experimented and published in the automated IED literature are not available for other researchers to replicate on their own data set and subsequently critique, improve or even compare with their own planned models. This very likely may be due to researchers considering that their models could be improved to a point of commercialization or alternatively suggest a lack of confidence on the generalizability of the model and thus keeping model details restricted and neural networks architecture explained in a general way. Such source code-sharing has been done in seizure detection algorithms.^69^ Any researcher who has published a model should avail their source code on a public repository to allow people to quickly test and validate the stated model performance on their respective private data sets. This will allow robust peer review. Such feedback can be provided back to the publishing author who can further fine-tune his model or be made aware of the model’s performance on different data sets to his. This would be easier than researchers sharing or requesting EEG data from other centres. If source code-sharing for cross-testing is not desired the next step would be standardized data sets to be made available in the public domain against which models from different research groups can be tested and compared. This however will not allow peer review of performance as model testing is carried out by the same authors and selection bias and selective reporting can still result. Thus, an open-source, code-sharing, mindset is definitely required for progress in this field to occur.

A note could be mentioned regarding classifiers being trained for whole EEG in contrast to individual IED marking. Data scientists and research labs interested in this metric should recognize that whole EEG classification will foreseeably remain the domain of human experts due to several reasons. With the advances in automated detection comes an understanding of the limitations of algorithms and the ethics surrounding their application.^70^ Hospital ethics committees or medical regulatory bodies will unlikely allow computers to make judgements on the labelling of an investigation as normal and abnormal which is to be extended without supervision to clinical care. As a parallel, most hospitals and health systems implement automated cardiac telemetry to screen for real-time diagnosis of arrhythmia. Even with the longer history of cardiac telemetry, its less complex signal characteristics and established role, human expertise and oversight is continuously needed so that unnecessary treatment is avoided. Despite this, cases have been reported of invasive interventions based on errant and artefactual automated telemetry results.^71^ Governance over automated assessment versus the clinician’s assessment of EEG will thus need to be closely monitored for the potential impact on treatment decisions and outcome. The focus instead should be on training, enhancing and improving the performance of IED classifiers to assist in marking and data reduction with a goal to speeding up the workflow of EEG laboratory and reviewing staff. It would be unwise to provide improved results on whole EEG classification, whereas the underlying goal of improvement desired in hospital practices is IED detection and automated marking.

### Enhancing automation

A great scope of research opportunities presents itself in this field. Once a sufficiently accurate or reliable computing model for a validated detection algorithm has been developed, several other opportunities will avail themselves to enhance such models. This could incorporate future work into automated classification of the various abnormal discharges into useful subtypes. This variation can be seen, for example, in genetic generalized epilepsy or symptomatic generalized epilepsy where several kinds of epileptiform abnormalities can present themselves either between or within a single patient’s EEG. The range of heterogeneity of discharges can include typical 2.5–6 Hz spike/slow wave, fragmented or localized spike or sharps, polyspike trains, polyspike/slow waves, paroxysmal fast activity^72^ and also atypical rhythmic or slow spike and wave. Similarly, in focal epilepsy, one can get different morphologic, topographic and periodic characteristics, including isolated or repetitive runs (brief and long trains), which could be rhythmic or semi-rhythmic and either confined to a limited topography unilaterally or could be bilateral or multifocal. All this may be further pursued by an upgraded algorithm based on the degree of channel involvement via some quantitative criteria. Voltage topographic maps and even more advanced source localization algorithms in high-density EEG could be integrated to easily pre-fill quantitative sections of reports for clinicians. Future wearable devices for seizure prediction can make use of IED burden or spike rate to predict an upcoming seizure. In one study, an accuracy of 92% for seizure prediction was noted using the spike rate threshold model.^73^ Predictor biomarkers currently being investigated could further allow potential predictability of pharmacoresistance in early clinical stages using large automated labelled data sets. Duration of epileptiform discharges, epileptiform burden and generalized polyspike trains, for example, are recent quantitative biomarkers associated with drug resistance.^74,75^ Persyst^76^ and the Encevis^64^/AIT team have been making progress in some of these domains and have commercialized their in-house AI algorithms. However, a systematic study and external validation will be required for more widespread use. This is currently being evaluated by the authors on a multi-centre data set.

## Conclusion

DL algorithms, despite success in seizure detection and clinical use, have so far failed to be implemented routinely for epileptiform abnormality detection in clinical care due to inconsistent and uncertain performances. Published algorithms remain doubtful as to their generalizability and are viewed with scepticism when it comes to clinical integration in the real-world setting. Clear protocols need to be devised regarding the description of training and testing data sets utilized, annotation methods, IED-benchmarking and more thorough performance evaluation and reporting of metrics. Open sharing of source codes after model publication should be promoted to allow cross-testing and independent validation of algorithms across data sets derived from different research and hospital settings. Despite the current stumbling blocks, a new era in clinical epilepsy diagnostics with automated IED detection is likely to emerge in the near future with DL methods at the forefront.

## Supplementary Material

fcac218_Supplementary_Data

## Data Availability

Data sharing is not applicable to this article as no new data were created or analysed.

## Abbreviations

AUC =: area under the curve
DL =: deep learning
ICU =: intensive care unit
IEDs =: interictal epileptiform discharges
IFCN =: International Federation of Clinical Neurophysiology
IRA =: inter-rater agreement
ML =: machine learning
TUH =: Temple University Hospital
