# Supplementary material for: Moving the field forward: detection of epileptiform abnormalities on scalp electroencephalography using deep learning—clinical application perspectives
Source: Brain Commun. 2022 Aug 29;4(5):fcac218. doi: 10.1093/braincomms/fcac218 (PMC9453433; doi:10.1093/braincomms/fcac218)
Supplement: fcac218_Supplementary_Data [file fcac218_supplementary_data.zip › Manuscript_original_submission.pdf]

OXFORD UNIVERSITY PRESS | Brain Communications

# Moving the field forward: detection of epileptiform abnormalities on scalp electroencephalography using deep learning. Clinical application perspectives

| | |
|---|---|
| Journal: | *Brain Communications* |
| Manuscript ID | BRAINCOM-2021-396 |
| Manuscript Type: | Review Article |
| Date Submitted by the Author: | 07-Nov-2021 |
| Complete List of Authors: | Janmohamed, Mubeen; Alfred Health, Depart of neuroscience; Monash University, Central Clinical School; The Royal Melbourne Hospital City Campus, Department of Neurology<br>Nhu, Duong; Monash University, Department of Data Science and AI<br>Kuhlmann, Levin; Monash University, Department of Data Science and AI<br>Gilligan, Amanda ; Epworth HealthCare, Neurosciences Clinical Institute<br>Tan, Chang Wei; Monash University, Department of Datascience and AI<br>Perucca, Piero; Austin Health, Department of Medicine and Neurology; Monash University, Central Clinical School; Royal Melbourne Hospital, Department of Neurology<br>O'Brien, Terence; Monash University, Department of Neuroscience, Central Clinical School; Alfred Health, Department of Neurology<br>Kwan, Patrick; Monash University, Central Clinical School; Alfred Health, Department of Neurosciences |
| Keywords: | Epileptiform abnormality, Automated detection, Deep learning, Machine learning, Electroencephalogram, EEG |
| | |

SCHOLARONE™ Manuscripts

# Moving the field forward: detection of epileptiform abnormalities on scalp electroencephalography using deep learning.

## *Clinical application perspectives*

Mubeen Janmohamed[1,2,3], Duong Nhu[4], Levin Kuhlman[4], Amanda Gilligan[7], Chang Wei Tan[4], Piero Perucca[1,2,5,6], Terence J. O'Brien[1,2], Patrick Kwan [1,2]

1. Department of Neuroscience, Central Clinical School, Monash University, Melbourne, Victoria, Australia
2. Department of Neurology, Alfred Health, Melbourne, Victoria, Australia
3. Department of Neurology, The Royal Melbourne Hospital, Melbourne, Victoria, Australia
4. Department of Data Science and AI, Faculty of IT, Monash University, Clayton, Victoria, Australia
5. Department of Medicine, Austin Health, The University of Melbourne, Melbourne, Victoria, Australia
6. Comprehensive Epilepsy Program, Department of Neurology, Austin Health, Melbourne, Victoria, Australia
7. Neurosciences Clinical Institute, Epworth Healthcare Hospital, Melbourne, Victoria, Australia

For Review Only

**Wordcount -** 7400

**Figures –** 4

**Tables -** 2

**References -** 71

# Abstract

The application of deep learning approaches for the detection of inter-ictal epileptiform (IED) is a nascent field, with most studies published in the past 5 years. Although many recent models have been published demonstrating promising results, deficiencies in descriptions of datasets, unstandardized methods, lack of demonstrable generalizability and variation in performance evaluation have made it difficult for these algorithms to be compared and progress to clinical validity. A few recent publications have provided detailed breakdown of datasets and relevant performance metrics to exemplify the potential of deep learning in IED detection. This review provides an overview of the field and equips computer and data scientists with a synopsis of Electroencephalography (EEG) datasets, background and epileptiform variation, model evaluation parameters, and an awareness of the performance metrics of high impact and interest to the trained clinical and neuroscientist EEG end-user. The gold standard and inter-rater disagreements in defining epileptiform abnormalities remain a challenge in the field, and a hierarchical proposal for IED labelling options is recommended. Standardized descriptions of datasets and reporting metrics are a priority. Source code sharing and accessibility to public EEG datasets will increase the rigor, quality and progress in the field and allow validation and real-world clinical translation.

For Review Only

# Introduction

Research in computer-assisted automated detection of inter-ictal epileptiform discharges (IEDs) transpired in the decades after EEG acquisition systems become available in clinical practice. The goal was to computerise detection of the 'sharp-transient' hallmark in epilepsy patients.[1,2] An early study pursuing this goal was in the early 70s,[3] where a now antiquated computer (PDP-12) was used to discriminate a waveform from a moving average derived from similar polarity amplitudes of 128 preceding waveforms. An indicator pulse was generated when the difference of a waveform amplitude reached a critical ratio. From that time onwards modern research has explored quantitative time-frequency algorithms as well as machine learning (ML) strategies to develop mathematical models with the intent to achieve reliable automated IED detection.[4] Deep learning, a relatively young field within ML, opens up a possibility to implement modern computing power to detect IEDs and improve workflow efficiency in EEG labs. Deep learning differs from traditional ML by using multiple hidden layers and has the advantage of automating feature extraction rather than manual feature selection, making the supervised aspect of training and learning much simpler.

A great deal of enthusiasm has been raised regarding deep learning outperforming expert specialists in health care diagnosis and clinical decision making, and a considerable amount of diagnostic, prognostic and treatment-based ML experimentation have been pursued and published across medical subspecialties. These studies continue to make headlines in various fields. As an example in skin lesion detection, the classification of lesions into melanoma versus benign nevi has shown convolution neural networks outperforming dermatologists in dermoscopic examinations.[5] In another landmark study for identifying and grading diabetic retinopathy using retinal fundus photographs, a deep learning neural network showed above expert-level sensitivity and specificity of over 90% in the detection of referable diabetic retinopathy and macular edema.[6] This required labelled imaging by 54 ophthalmologists on a dataset of 128,000 images for training and validating and testing in a subsequent dataset where it outperformed health experts.

For Review Only

Such examples of remarkable success however should not be prematurely taken to conclude that ML in health has reached an implementational level in real-world clinical practice. When the above promising retinal classification model was deployed in a real-world prospective study in Thailand several impediments were identified affecting system performance.[7] 21% of the retinal photographs were rejected by the algorithm as they did not meet the system's high standard for grading even when they were of adequate quality to be graded by the human visual eye. Real world clinical data on the ground is frequently affected by a diverse range of technicalities which health experts have to regularly deal with and this is particularly pertinent in the EEG and epilepsy world.

For Review Only

Deep learning in the EEG field covers a broad scope of research including epilepsy, sleep diagnostics and brain-computer interfacing.[8,9] Within clinical epilepsy itself, ML approaches have been investigated for seizure detection,[10,11] seizure prediction,[12] epileptiform detection,[13] epilepsy imaging, genetic mining and classification, medical[14] and surgical treatment decision-making and clinical outcome prediction.[15] High discriminative abilities have been asserted in these varied fields; however there remains an uncertain perspective of real-world implementation and generalizability.

A recent study related to EEG IED detection employed a 10-fold cross-validation method on over 13,262 IED candidate waveforms.[16] A very impressive AUC of 0.98 of IED detection, was cited for a deep learning model developed and termed as SpikeNet. Additionally, an AUC of 0.847 was also reported for classifying whole EEGs using a binary classifier trained using 10 extracted features. The model reportedly outperformed fellowship-trained EEG experts to detect individual IEDs. This number however needs to be contextualized. All data was obtained from a single centre including training and test dataset and an external out-of-hospital test dataset was not employed. An epoch-based graphical user interface (GUI) point and click format (Neurobrowser) was employed which did not allow contextualizing the EEG for the blinded comparators beyond the window shown. The overall inter-rater reliability in this particular study for these blinded reviewers agreeing on candidates as spikes was only

fair with (Gwet κ) of 48.7. Most importantly the source-code for this model has not been available on public repositories to validate on external independent datasets. This external validation limitation in ML is well known.[15]

This review summarizes some of the perspectives of clinicians who have provided clinical support in ML IED detection in collaboration with data scientists via EEG data obtained from tertiary epilepsy centres in Melbourne. The article will allow data scientists and ML researchers entering the IED detection field to quickly understand the basic nature of the EEG data used in epilepsy management, challenges they will encounter upon embarking their journey and recommendations on moving the field forward.

**Search strategy and selection criteria**

References for this Review were identified through searches of PubMed with the search terms “inter-ictal, “epileptiform”, “spike”, “deep learning”, “automated software”, and “epilepsy” from 2010 until March, 2021. Only papers published in English were reviewed. A recently submitted but as yet unpublished systematic review from our group synthesizing methodological details and performance of recent deep learning papers was used. The final reference list was generated on the basis of originality and relevance to the broad scope of this Review

# Why research in this field and limitations

The digital era has opened itself to automating tasks requiring human efforts, especially those which are repetitive and time-consuming. This pursuit has been embarked to make hospital workflows more efficient. A routine EEG recording of 30 minutes usually takes anywhere between 5 minutes to an hour (median 13 minutes) to be visually assessed and reported by an epilepsy specialist, depending on various factors including presence of abnormalities, length of the EEG and artefacts present.[17] This time can also be increased or



For Review Only

decreased based on the setting of the EEG. In the ICU setting, 24 hours of abnormal continuous EEG being reviewed for only seizure identification required a median of 44 (+/- 20) minutes in a retrospective review of conventional review versus quantitative EEG comparator study.[18] In contrast, a ML algorithm can take minutes to label and provide prediction labels for a 24-hour EEG. A recent paper showed an average computational time of 7 seconds to label signal lengths of 1 hour.[19]

Identification and interpretation can take longer during manual review for more difficult and complex EEG data. An example would be intracranial data of a patient with a complex epileptogenic zone and several dozens to hundreds of electrode contacts resulting in a vast number of channels to review. Inconsistent labelling is also common in practice as different EEG technicians and clinicians use different approaches and terminology in marking data. A successful computer-assisted detection would theoretically vastly reduce the time and improve quality of labelling currently being done manually by EEG scientists, technicians and clinicians.

The most concerning limitation of implementing ML in future workflows would be misreporting of an EEG by an overseeing clinician, in particular a non-expert epileptologist, biased by the automated program. Every EEG has variation, and no model will ever result in a 100% accuracy. An EEG may be reported as positive when not, resulting in unnecessary and even harmful treatments being implemented, and conversely false negatives may delay treatment with the potential to cause harm to patients. This problem has also been often noted in global clinical practice, outside the expert neurophysiology community and addressed in a series of articles in 2013 appearing in Neurology. In a survey of 47 trained neurophysiologists, during the annual meeting of the ACNS in 2010, many noted coming across misread EEGs and 38% encountering them frequently[20]. In a more recent study in India[21], 1862 EEGs were prospectively performed to identify the prevalence of benign epileptiform-like-variants (BEVs). Under recognition and misreporting were common in the neurology community. Amongst 101 subjects whose previous raw EEGs were accessible,

For Review Only

30% were noted to be misinterpreted as epileptiform abnormalities. In the hands of inexperienced EEG readers, an automated detection program may confound and potentially worsen misreporting. Several recommendations and guidelines across the epileptology literature[20,22,23] have been made to reduce this risk and efforts are in place to increase the training, teaching and reporting of EEGs.

**Table 1** *Pros and Cons of future computer-assisted detection in EEG laboratories*

**Pros**

- Super-speed labelling and substantial data reduction leading to faster workflows
- Substituting unavailable expertise in low-resource countries
- Artificial intelligence is purported to have the potential of better results than traditionally trained experts.

**Cons**

- Missed true epileptiform discharges (false negatives) with potential to delay treatment.
- Exaggerated labelling of artefacts as abnormalities (false positives)
- Reduction of job and learning opportunities for EEG scientists and epilepsy trainees.

For Review Only

# Overview of scalp EEG datasets available for machine learning

A wide array of EEG recording types can be retrieved from hospital based EEG servers (See, Figure 1). A scalp outpatient routine EEG is the simplest of the raw EEG datasets available and is usually recorded in a 10-20 electrode configuration, with or without ear electrodes. Routine EEG recordings are frequently done in rested patients who are not in an unwell clinical state and can generally, at most times, follow instructions. The quality of the

EEG signals would be amenable for machine and deep learning as recording technicians in real-time are able to improve the quality of the signal recording and annotate important segments for a further clinician's review. This has been a common dataset used in deep learning literature. Routine outpatient EEGs typically range between 20-30 minutes and sometimes a more prolonged 1–3-hour sleep-deprived or non-sleep deprived EEG may be requested by the clinician overseeing the patient's care.  Sleep deprived EEG similarly provide good quality recordings given artefacts from movement and muscle are considerably reduced during sleep, and a marked surge in epileptiform abnormalities is seen in both focal and generalized epilepsy during sleep.[24–26] Sleep however presents a different overall background from which the epileptiform abnormality emerges, and the epileptiform abnormality can present different morphologic characteristics and of briefer duration in the case of genetic generalized epilepsy.[27]

For Review Only

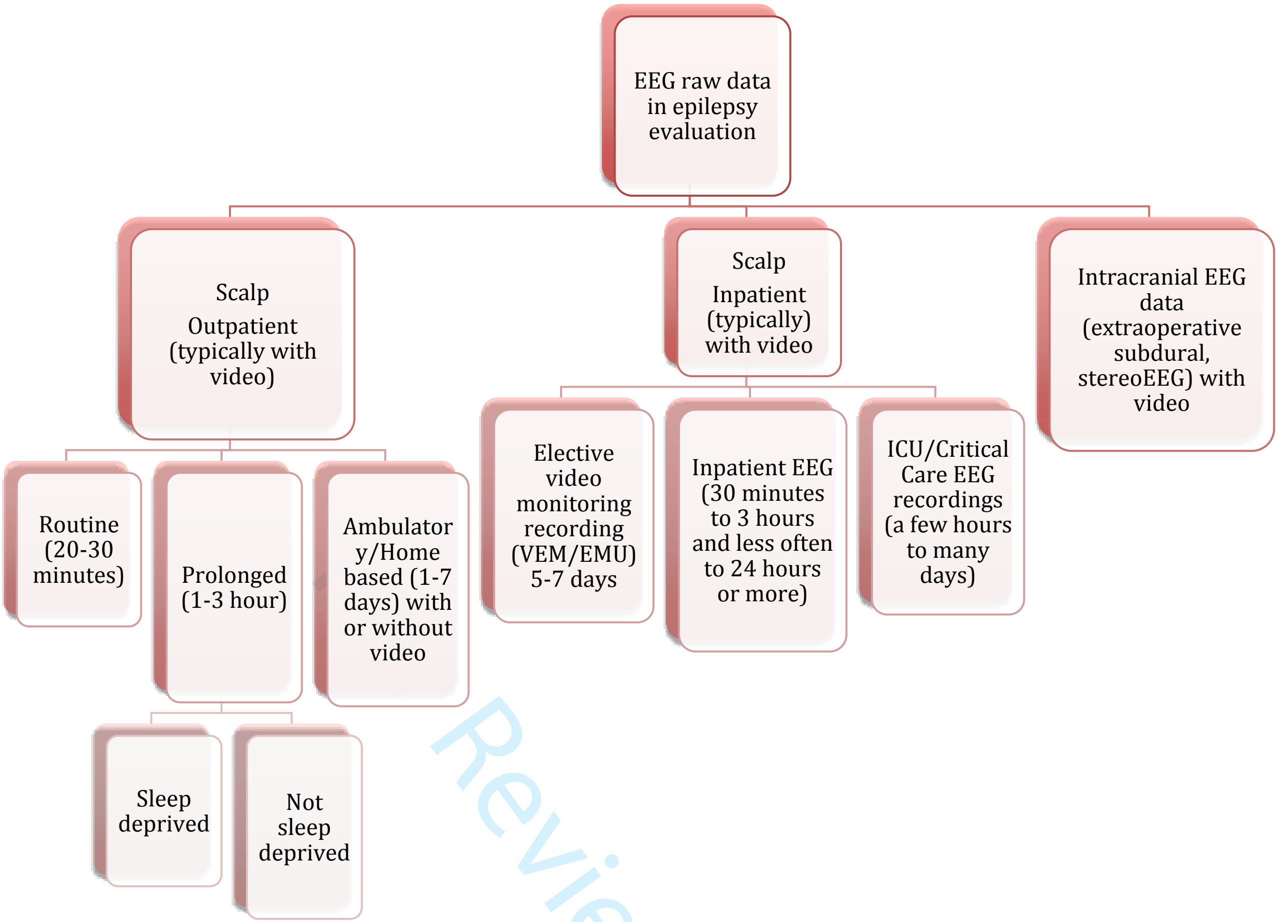


*Figure 1 The structure of data available from hospital-based EEG servers*

Review Only

A downside of routine EEGs is that given their shorter duration they are less likely to have abnormalities to gather for the training dataset. Major tertiary referral centres however would still have several hundred to thousands of routine outpatient EEGs which are abnormal and containing epileptiform abnormalities stored on their servers depending on the protocol of archiving used and format compatibility with modern software. In a hospital, an EEG recording can also occur in a ward-based inpatient setting, a multiple-day elective video monitoring setting or the critical care setting.  Video EEG recordings of patients who are electively admitted for a multi-day recording would be intermediate in quality. Scientists are able to correct loose electrodes and aim to reduce artefact contamination, improve impedances and educate the inpatients to aim for better technical recordings.  Sleep

background is also available, and video is always available to correlate abnormalities for review. Here the number of electrodes can differ depending on the purpose of the elective admission. Recordings for surgical localization regularly have additional sub-temporal electrodes. Sometimes symmetric or asymmetric higher density electrode coverage in addition to the standard 10-20 electrode placement system may be carried out in different regions of the brain which introduce variation. Such video-EEG recordings can easily be processed to a 10-20 format for further data processing.

Lesser quality datasets would include ambulatory EEG[28,29] recorded when patients are up and about at home, introducing large movement and myogenic artefacts and where an overseeing scientist is not reviewing the record until the leads are removed the next day or at the end of recording duration. Perhaps, most challenging of all would be critical care patients where electrical interference from surrounding equipment causes significant artefacts, electrodes may be placed in non-traditional positions or excluded due to craniotomies and the background may be confounded by sedative medications or the underlying brain insult. Prolonged ICU continuous EEG is often performed in hospitals for monitoring seizure activity of critically unwell patients.[30]

# The nature of EEG background and epileptiform discharges

Given data scientists, and those involved in ML training and testing, are not clinicians, they may lack a ‘hands on’ understanding of the diversity and dynamic nature of both background and epileptiform discharges in an EEG. This variation has to be understood before embarking on the ambition of a universal IED detection model. Figure 2 demonstrates sample EEG epochs of epileptiform variation in a genetic generalized epilepsy dataset.



For Review Only

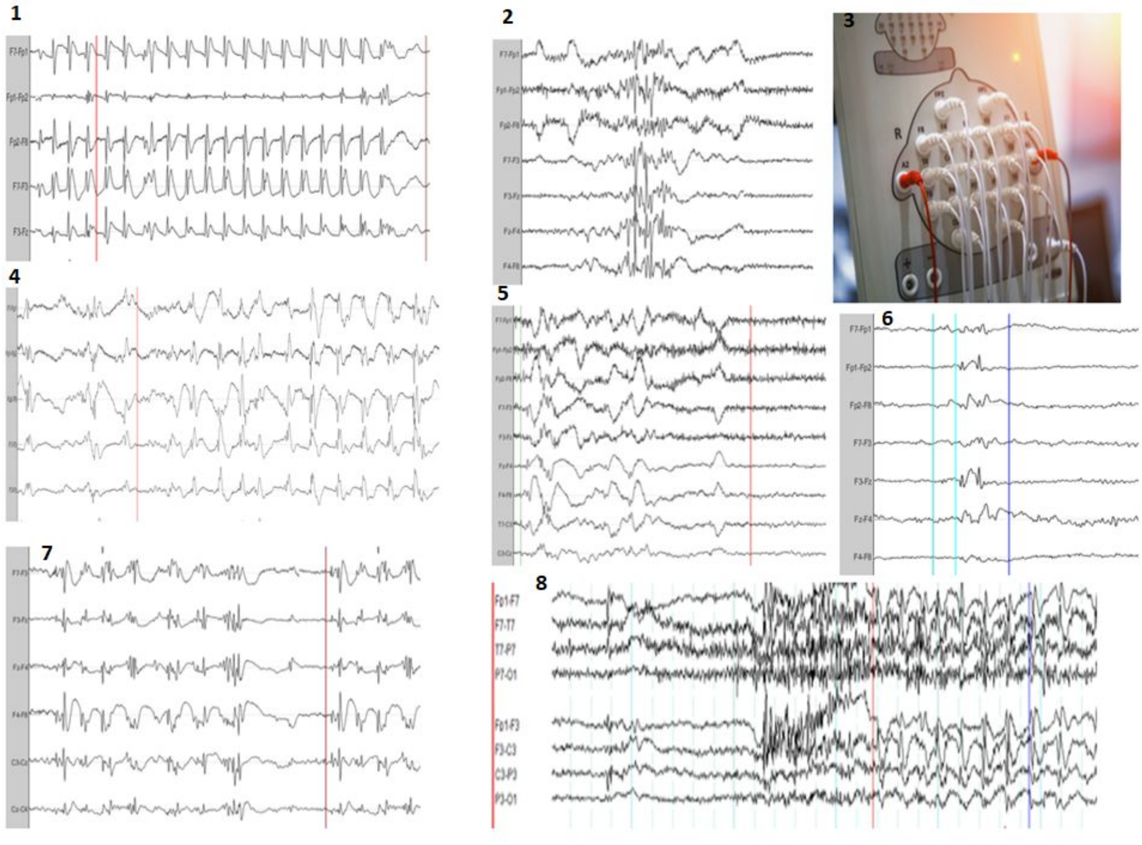


*Figure 2 Epileptiform variation in GGE EEG datasets (From Left to right) 1. Classic 3 Hz spike and wave on transverse montage, 2. polyspikes with EMG artefact in frontopolar channels and eye movements, 3. EEG amplifier for 10-20 recording, 4. slow spike/wave on transverse montage, 5. mild EMG affecting frontal channels with embedded small spike and waves and irregular slow waves, 6. fragments on transverse montage, 7.polyspike/slow waves on transverse montage, 8. marked EMG artefact confounding epileptiform abnormality in temporal and frontal channels on longitudinal montage*

The normal background of an EEG is dynamic and can be divided into normal awake, drowsy and sleep stages.[31] Background frequencies are slower and less dynamic in encephalopathic patients or those with developmental delay and neurodegenerative conditions. There can even be an association of background frequencies with age. Paediatric EEG has a much more complex range of normal background while older patients

demonstrate slowing of the dominant posterior alpha activity.  Simple state-occurrences like eye closure can also modulate the background. A wide multitude of technical issues and artefacts can add to tremendous variation in the background during the awake state and some of these can resemble epileptiform abnormalities.[32] These can occur in both the awake and drowsy state. Several background variants of the normal EEG can occur including alpha variants (fast and slow) and posterior slow waves of youth. Further benign epileptiform like-variants can occur in the EEG during awake and drowsy state and may include Benign small sharp spikes (BSSS or BETs), wicket waves, 14 and 6 positive spikes, and 6-Hz phantom spike and wave.[31,33,34] Physiologic changes in the drowsy and sleep record include attenuated posterior rhythm, central theta, V-sharp (vertex) waves, large K-complexes, spindles, arousal patterns,  positive occipital sharp transients (POSTs) as well as temporal or diffuse rhythmic theta and high-amplitude delta slowing.[35] Detection models may confuse some of these morphologies with epileptiform discharges (See, Figure 3), especially those discharges showing rhythmic sharply contoured waves forms or even delta duration slow waves, such as the kind seen in spike/slow wave.[36] Similar to dynamic changes in background which characterize a normal or abnormal EEG, there is no uniformity in epileptiform discharges within and across patients. Epileptiform discharges can vary in duration, morphology, periodicity, topography and can be modulated by either state changes or other provocative manoeuvres. The diversity of epileptiform discharges can include spikes (20-80 ms), sharp waves (80-200 ms), spike-slow wave, sharp-slow wave, polyspikes, polyspike-slow waves as well as fast activity associated with the aforementioned. These can be located, within one subject, in one region or in two or more regions either in one hemisphere or bilaterally. When these engage bilateral networks, they are referred to as generalized epileptiform abnormalities and when confined to one hemisphere in a few electrode sensors as focal abnormalities. They can occur as isolated transients or be part of a sequential train or run which can be periodic[37] or quasi-periodic.  In some patients these frequently recur through the duration of an EEG recording but may only occur only

For Review Only

occasionally in briefer recordings. They can occur in combination with any of the background states mentioned above.

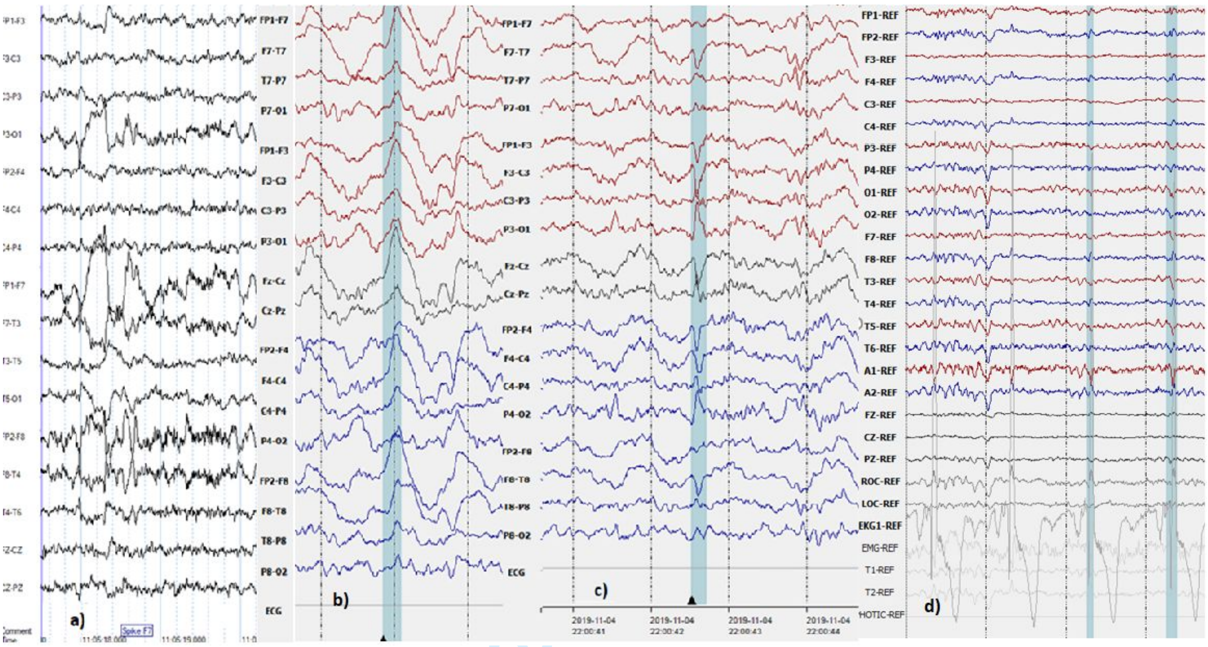


*Figure 3 IED mimics a) Eye and EMG resembling IED mimic detected by algorithm. b) high amplitude slow wave in Stage 3 sleep causing false positive c) v-wave mimicking sharp wave and labelled as abnormal by algorithm d) ECG artefact picked up as runs of IEDs*

eview Only

Sleep modulates and accentuates the occurrence of epileptiform discharges (Seneviratne et al. 2020) in focal as well as in generalized epilepsy and so does provocative manoeuvres which in different epilepsies may include photic stimulation, [38] hyperventilation or even things like visual, audio or cognitive tasks.[39,40] An ambitious all-spike (universal IED detecting) deep learning model should have undergone training with a vast amount of EEG capturing the majority of this variation. The differences further are confounded by EEGs available from different settings where different noise levels will be present. To reiterate, an outpatient ambulatory EEG recording is not equivalent to a routine resting EEG in terms of background quality and both of those will be different to EEGs acquired in a critical care EEG.

# Performance testing of Models

## Inter-rater agreements and the gold standard comparator.

One of the main concerns in current ML studies is the lack of an unambiguous framework of what the gold standard or 'ground-truth' is for determining the accuracy of the final computing model.[41] Given the consistent real-world underperformance in the reliability of computers versus humans in EEG IED discretion, the reference standard today remains visual review and classification by a trained epileptologist or clinical neurophysiologist. This presents some limitations as inter-rater reliability amongst EEG readers has been controversial due to the perceptual phenomenon and probabilistic art of reading spikes.[42] For decades the question of inter-rater (IRA) agreement has been investigated in parallel with the question of computerized detection. The FDA requires three electroencephalographers in the annotation process for approval of an algorithm[43]. Some authors have directly looked at inter-rater reliability and clearly specify the number of EEG raters selected for an internal criteria of a definite IED.[16] Halford (2009), mentions at least four different agreement criteria used by ML-authors for referencing actual IEDs in his review paper including concordance between all raters, a cut-off number of raters from the whole group, reconciled rating amongst reviewers and some papers using only one rater to define the gold standard. Expert pooling has been found to be better and larger group sizes from 3-10 have been reported to be ideal yet judiciously selecting expert electroencephalographers (EEGers) for IED annotation research projects may reduce the need for this number[43]. Other researchers simply mention the use of a confirmed video-EEG 'epilepsy' diagnosis to validate their annotations without elaborating further. This however does not necessarily mean that all



For Review Only

labelled IEDs should be considered gold standard as epilepsy patients habitually can have benign waveforms or IED mimics at the same time.

Although the above may lead to a perception that IRA amongst EEGers is imperfect and unreliable, this is not entirely accurate. Several studies have shown moderate to substantial inter-rater agreements. Some studies report higher Gwet or kappa as well as high performance of blinded clinical experts compared to an unblinded gold standard [44–46].Inter-rater agreement in ‘whole EEG’ categorization remains high given low-perception spikes are contextualized by EEGers before concluding the report as normal and abnormal. A limitation of poor or fair IED agreement studies is that reviewers are blinded to clinical context or deal with very short segments of data when tested.[47] Most real-word EEG review of waveforms requires an awareness of patients' age, compliance with recording instructions, technical quality, sedative agents, pharmacologic agents, previous EEG characteristic, clinical context and the conscious state of the patient during recording.

A recent paper provides a good benchmark and sheds light on what can be used as a gold standard. Kural et al[44] assessed six criteria that are used to determine what is or is not an epileptiform abnormality to assess inter-rater variability amongst clinicians for each feature criteria and assess the IFCN criteria as a whole for validity. In the study, they used a strict methodology to confirm an IED. It required two reviewers agreeing that the candidate waveform was a sharp transient and furthermore additional criteria of a patient not only to have confirmed epilepsy but of the selected transient being concordant with the patient’s recorded seizure and location as expected in that syndrome or focality (inter-ictal, ictal and syndrome correlation). The clinical context was thus extensively incorporated in the decision-making of what is or is not a spike thus setting an acceptable benchmark for gold standard for the purposes of that study. With that gold standard other methods of defining epileptiform were evaluated. Blinded reviewers utilizing 4 or 5 of the 6 IFCN criteria together provided a strong accuracy of the waveform being labelled as epileptiform with accuracy levels of 91% (95% CI 83.6-95.80) and 88% (95%CI 80-93.6) against the gold standard. Furthermore,



For Review Only

experts solely using their clinical experience with no protocol method and simply consensus provided a 92% (95% CI 84.8 – 96.5) accuracy. It is important to note that all these experts were blinded to the original two reviewer unblinded gold standard assessment. The six IFCN criteria used included the morphology of spikiness or sharpness, asymmetry of ascent-descent slope, duration difference from background, an after-going slow wave, background disruption and a concordant voltage map.

## Proposed Hierarchy for gold standard epileptiform detection

With this above mind, several ways can be used to formulate a gold standard for the deep learning experiment in a hierarchical order. In the most reliable and resource consuming situation experts (even a few) decide that each spike or sharp wave in relevant time epochs is an epileptiform abnormality having awareness of the context of the patient's profile (clinical history and imaging) and have access to longer EEG recording or at least more than just short epochs, including ictal patterns to correlate if accessible. EEG reviewing and reporting, especially for inpatient EEGs, eventually considers all clinical comments regarding why the EEG is performed. This criterion may not be practical for big data research projects. Secondly, despite the above-mentioned limitation, it can be stated with some certainty that that even very few sufficiently trained electroencephalographers utilizing the 4 or 5 of the 6 IFCN criteria mentioned above, preferably with basic awareness of the context for the EEG being labelled, would be a validated gold standard. The third method would be epilepsy experts either contextually or blindly marking via 'experience' without adequate exposure of the entire EEG and clinical context of the respective EEGs. Due to sub-par inter-rater concordance frequently cited in the literature, the third method remains controversial in terms of how many people should agree. Occasional papers however continue to show an adequate level of expert agreement, even when this method is employed.

If there are limitations in obtaining epilepsy experts to annotate, a layered approach can be used where a less rigorous lesser man-power method is used to annotate the training

For Review Only

dataset and a more rigorous consensus method used to annotate the testing dataset. This will ensure that the testing results provided in the study have been compared against the best available gold standard in that centre.

## Annotation standards

Recent literature on deep learning IED detection do not usually elaborate on the precise annotation protocol used for the supervised learning process.  A six-way labelling classification of epilepsy EEGs is employed by the Temple University group which has availed their public dataset on the internet.[48] Proper annotation labelling may be important for the performance of the algorithm. An abnormality can be marked using a single marker or a start and end marker. The windowing method employed for training and testing purposes for IED detection may incorporate partial normal segments in the windows designated as abnormal containing epileptiform abnormalities. If only one marker is used it can be placed at various points along the discharge most frequently at the negative peak of the spike portion. No strict rigor can be employed here due to the vast heterogeneity of the way transients and prolonged discharges appear. Even when a more laborious 'start' and 'end' markers are utilized there is frequent inter and intra-rater variation from our centre's experience in labelling as abnormalities often do not have clean onsets and offsets. A decision may be made to annotate first spike onset however spikes may terminate before the discharge has ended in the case of generalized epileptiform abnormalities.[49] Conversely, discharges may emerge with some abnormality in background or rhythmic slowing before showing clear spike morphology.  In the case of stereotyped repeated focal transients, the onset and offset may be easier to define. An annotation marker may be generic with an instant timestamp without regard to the channels involved or it may be specific and labelled according to the specific channels involved. The TUH events public corpus has made a tremendous effort to label abnormalities based on specific channels involved and may allow



For Review Only

more precise abnormal signal input for the subsequent learning process.[50] These annotations of the datasets however will need to be systematically validated.

# Metrics evaluation

Utilising sensitivity, specificity and area under the curve of each model does not always translate into useful clinical assessment. There remains a challenge with reporting metrics in regard to IED deep learning models and the most clinically useful metrics are underreported in the currently existing literature. Many authors are needlessly focused on ‘accuracy’ or ‘AUC’ which represent area under a ROC curve, comparing sensitivity and 1 – specificity for various thresholds. Accuracy is assumed to be the most intuitive measure to understand, given it represents the proportion of correct predictions compared to true gold standard observations. Both accuracy and to some extent AUC as understood by data scientists in the field, unless contextualized with other metrics, have limited significance for real-world clinical use, given there will always be hundreds to thousands of segments (usually 1-2 second windows) as negatives. Some authors have utilized the concept of balanced accuracy (BAC) which is the arithmetic mean of sensitivity and specificity and provides a better perspective than crude accuracy (ACC). Far more common than accuracy, recent deep learning models consistently report over > 0.9 or 90% AUC[16,51–55] which may appear to be remarkable however will also be challenging models to implement in practice and should be interpreted with caution. As an example, an EEG of 30 minutes has 900, 2-second windows or 1800, 1-second windows. A routine EEG in the awake state can have a variable number of epileptiform discharges, ranging from 0 to typically a few dozens. A model can be stated as being 90% ‘AUC’ and yet be unreliable from the clinical perspective. This will occur if the normal windows for the majority part were correctly predicted even if the abnormal discharges, which occupy very brief lengths and occur sparsely, were all mostly missed. The only tuning of the model to give a high accuracy would be to reduce the number of false positives which could be attained by raising the detection or perception threshold (low

For Review Only

sensitivity setting). True negatives in such a model will be high in both the numerator and denominator, falsely giving an "accurate performance". An AUC does not always solve this problem as both a high sensitivity and specificity do not necessarily address the issue of false positives. This problem of imbalanced dataset during ML training occurs when normal background input vastly exceeds abnormal windows with a ratio of up to 1:1000[52]. Data augmentation methods have at times been incorporated to increase representation of the spike minority class using oversampling techniques.[56,57]

When evaluating a deep learning model, several other metrics therefore have to be taken into account and few metrics are helpful as standalone measures to give a perspective on success (See, Table 2). Precision reports true positive IEDs in the entire set of predicted IEDs. It represents the positive predictive value in clinical terms. This coupled with sensitivity represent a better measure than specificity and AUC. Class imbalance as described above skews specificity TN/(TN+FP) and other measures dependent on it like AUC. Amongst other parameters of high utility is the F1 score which provides a weighted average of both sensitivity and precision and the AUPRC which is the area under precision-recall curve (AUPRC). The F1-score weights the two most important variables and will take into account false positives and false negatives without contaminating or exaggerating performance with the imbalanced true negatives. False positives per minute is a simple and informative metric which provides accurate insight into performance when full length EEGs are evaluated in the test dataset.

**Table 2:** Performance metrics commonly used in machine learning studies

For Review Only

## Metrics of clinical utility for IED detection

- **Sensitivity**: Proportion of true gold standard IEDs correctly detected
- **Precision:** The proportion of true marked gold standard IEDs to all machine predicted positive labels. (True Positives)/(True positives + False positives)
- **False positive rate:** Rate of false positives which were not classified by the gold standard as IEDs typically reported in per hour.
- **F1-score –** This takes into account the two most relevant metrics of precision and recall.
- **AUPRC** - Area under the precision-recall curve (AUPRC) which differs from the area under the ROC curve. A model achieves perfect score when it identifies all epileptiform abnormalities without marking normal or benign abnormalities.

## Metrics of limited clinical utility in isolation

- True negatives, specificity, accuracy and AUROC (area under ROC curve).

For Review Only

If per channel evaluation is desired modifications to appraisal will be required in a mixed unselected dataset as generalized epileptiform abnormalities or spikes with broad fields will show preferential biasing compared to localized spikes. From the clinicians' perspective single channel annotation would be tedious to review, present redundant data and pose difficulty in evaluation when many channels are involved. A single timestamp even for generalized or broad field focal abnormalities would serve the intended purpose of automated detection as a screening tool.

Evaluation may also further be confounded by the division of data sets used for training. To measure how well a model classifies a randomly given set of windows, a researcher may use only the normal EEG testing dataset for calculating false positives whilst ignoring abnormal EEGs with an errant understanding that abnormal EEGs should not be used for evaluating false positives. This may occur if training data was split separately with abnormal windows derived only from abnormal EEGs and normal windows from normal EEGs to avert the possibility of missed IED labels in the preparation of the training dataset. Given this suitable and correct dataset preparation criteria, a researcher may then extend this logic to

test false positives only on normal EEGs and false negatives (missed IEDs) on abnormal EEGs with the perception that error margin of any potential unlabelled IEDs in the abnormal EEG would not allow accurate performance determination of the algorithm. This testing protocol does not make any intuitive sense and testing performance should be on a mix of combined normal and abnormal EEG dataset with performance measures evaluated and reported for the whole entire dataset. If concerns over accuracy of labelling in the test datasets exist, all manual annotations carried out on the test dataset EEG should be carefully reviewed with added clinical reviewers to ensure that all abnormal and normal segments have been rigorously identified. In the case of highly active prolonged abnormal EEGs the recording can be trimmed to reduce margin of error for the manual annotator instead of using dozens of hours. Furthermore, manual review and validation of the automated spike labels should also be performed to ensure any extras detected by the machine learning are false positives and not detected but unlabelled true positive IEDs.

## How should an IED model fit for clinical integration be reported – focus on the metrics

Details on how the analysis and performance statistics were calculated should be elaborated in forthcoming deep learning studies. Were brief epochs or entire lengths of EEGs evaluated in the test dataset to decide on prediction accuracy? Accuracy/AUC calculated on IEDs in a substantially imbalanced real-world dataset of whole EEG recordings is different from Accuracy/AUC calculated on a dataset limited to segmental review or epochs with an attempt to balance normal and abnormal segments in the test set. Segment or epoch based metric calculation would make AUC and accuracy valid only for that specific dataset however would not allow inference on longer EEG durations in a real-world setting. Selection bias can also be introduced into the epoch-based methods as noise-free segments with better



https://mc.manuscriptcentral.com/braincom

For Review Only

technical quality, uncontroversial epileptiform discharges and more normative backgrounds with less complexity can be chosen by the dataset retrieving team. The problem of imbalanced dataset has to be tackled in this field as epilepsy data will always have the vast majority >95% or more of its signal to be normal background apart from a few outlier intractable epilepsy patients who have frequent, near continuous or continuous epileptiform abnormalities interspersing background. The benchmarking test dataset must therefore be an imbalanced dataset if any real-world clinical utility is to be desired.

Another question which may arise is how were discrepancies between IED discharge duration and windows addressed if the performance is validated using manual visual review of the computer labelled marker? A single discharge of just over 2 seconds defined from start to end may have 3 'correct predictions' instead of one given the overlap of three 1-second windows to abnormality length. This may over exaggerate performance measures for discharges which are prolonged beyond the windowing duration as multiple markings may be made and all deemed as true positives. To simplify, an EEG may hypothetically have two spike/wave discharges of different durations - one of 0.5 seconds and one of 10 seconds. A detection algorithm using 1 second window segmentation may detect all the windows of the 10 second discharge and miscategorise the 1 window overlapping with the 0.5 second discharge. A sensitivity of 91% (10 out of 11 windows detected) will be reported based on windows evaluation however if raw spike/wave count is used then a sensitivity of only 50% (1 of 2 spike/wave discharges detected) will be reported. Similarly precision and other metrics relying on false and true positive definitions will be affected. A false negative (missed epileptiform abnormalities) on the other hand may be deemed as one missed epileptiform abnormality during manual review unless the windows are correlated to the discharge duration. Redundant markings between pre-labelled start and end marking of a single contiguous discharge should therefore not be counted as true or false predictions and should not influence metric calculations. An explanation of how the manual labelling was carried out and how performance was evaluated would enable more rigor in future studies,

For Review Only

especially dealing with EEGs containing prolonged trains or where short window duration is used.

## Whole EEG Classification

In this area of interest, some authors have reported on IED-free vs not-free to categorize whole EEG classification.[16,53,55] This may implement a IED rate threshold to categorize EEGs into normal vs abnormal which differs from criteria used in real world classification by epileptologist. Clinically one unequivocal epileptiform abnormality suffices to classify an EEG as abnormal. If EEGs were sorted from clinical reports, caution needs to be adopted given other EEG features, most notably focal slowing or indeterminate findings, can lead to the classification of an EEG as abnormal without an epileptiform abnormality. Textual mining of reports for dataset retrieval should specifically require the presence of epileptiform abnormalities and the conclusion of the report should be ascertained. Sensitivity and specificity calculations are easier to calculate for whole EEG classification as actual positives and actual negatives are easier to define without the challenge of defining windows in relation to IED durations. Inter-rater agreement on whole EEG classification is higher than for individual IEDs.

# Future directions and overcoming the IED challenge.

In seizure detection, low false alarm rates (<1/hour) and high detection rates (70-80%) have been achieved [58] and software are operational in some hospitals for seizure alerting, detection and continuous monitoring.[59] On the other hand, despite more than 50 years of

For Review Only

study in this study area, commercial or open-source software have not become pervasive in clinical use for the detection of IEDs on EEG recordings despite the immense benefit to time and labour challenges in an EEG lab. A recent commercialized spike detection software trained using deep learning algorithm, Encevis Solutions[60] (Austria), sensibly uses clustering to overcome the high rate of false detections 112/h for a high sensitivity of 89%.[61] Persyst 13 has been reported in one study as non-inferior in performance to senior EEG technologists[62] at a low perception predictive setting (high sensitivity setting) but was found to have much higher false positive rates at various perception thresholds compared to board certified EEGers reviewing Epoch based transients.[63] It remains to be seen how well the software will perform on more varied, larger and longer unselected EEG datasets using reliable gold standard. There remains significant scepticism amongst EEG technicians and clinicians as to the benefits of available software accurately guiding the process of capturing and labelling spikes on scalp EEGs and subsequently quantifying IEDs let alone precisely making a judgement on classification of a scalp EEG into normal or abnormal (IED free vs IED-EEGs).

The scepticism and difficulty in readily available deep learning algorithms for computer assisted clinical EEG reporting has been due to a great number of barriers. The wide heterogeneity of methods, statistical approaches and reporting in current literature introduces difficulty in comparison of models. The models may appear accurate in a single centre dataset but their applicability to multiple datasets is more challenging. Standardized descriptions of datasets and reporting metrics is essential remains a priority in this field.

A recent exemplary paper provides details to properly understand a deep learning publication of IED detection. Adequate description and division of EEG data was provided, epilepsy syndrome details, method and algorithmic details and most importantly comprehensive performance metrics results.[19] A framework for relevant details in a deep learning IED detection study is provided in Figure 4. For the dataset, there should be a clear explanation of the recordings being either scalp or intracranial, the environmental setting in which the EEG was performed, and the breakdown of the type of EEGs used for both

For Review Only

training and testing datasets. Extended labels are preferred to single timestamps during manual labelling otherwise the IED discharge will be assumed to be the window size by the performance evaluator to enable calculation of relevant metrics. If combinations of heterogenous recorded EEG data in different inpatient and outpatient settings are used, the proportion of different respective EEG types implemented in both the training and testing dataset should also be described. Electrode configurations used and channel derivation from electrode montaging should be mentioned as these can introduce some differences in signal characteristics. Average referenced signals can be different depending on vertex or ear electrode referencing or all-electrode averaging which can be different from bipolar or transverse derived signals. The montage ultimately chosen for testing and training may introduce some variation in the derivative signal and may not translate well into a test dataset using a different montage configuration for signal derivation. As an example, waveforms may appear sharp and phase reversing on bipolar whilst not appearing different from the background in an average montage. Similarly averaging can sometimes bring out waveforms which undergo differential voltage cancellation in bipolar montage due to equal strength amplitudes. Very few publications provide information on epilepsy type, syndromes and nature of discharges and whether the bulk of the abnormal discharges or the majority background used was derived from awake or sleep state or in what estimated proportion. This can be important as a dataset used to train a focal epilepsy model may not be appropriate for a generalized epilepsy test dataset. Further, some models may work well on awake background but present false positives in sleep EEG due to low frequency waveforms being confused with slow wave abnormalities.

For Review Only

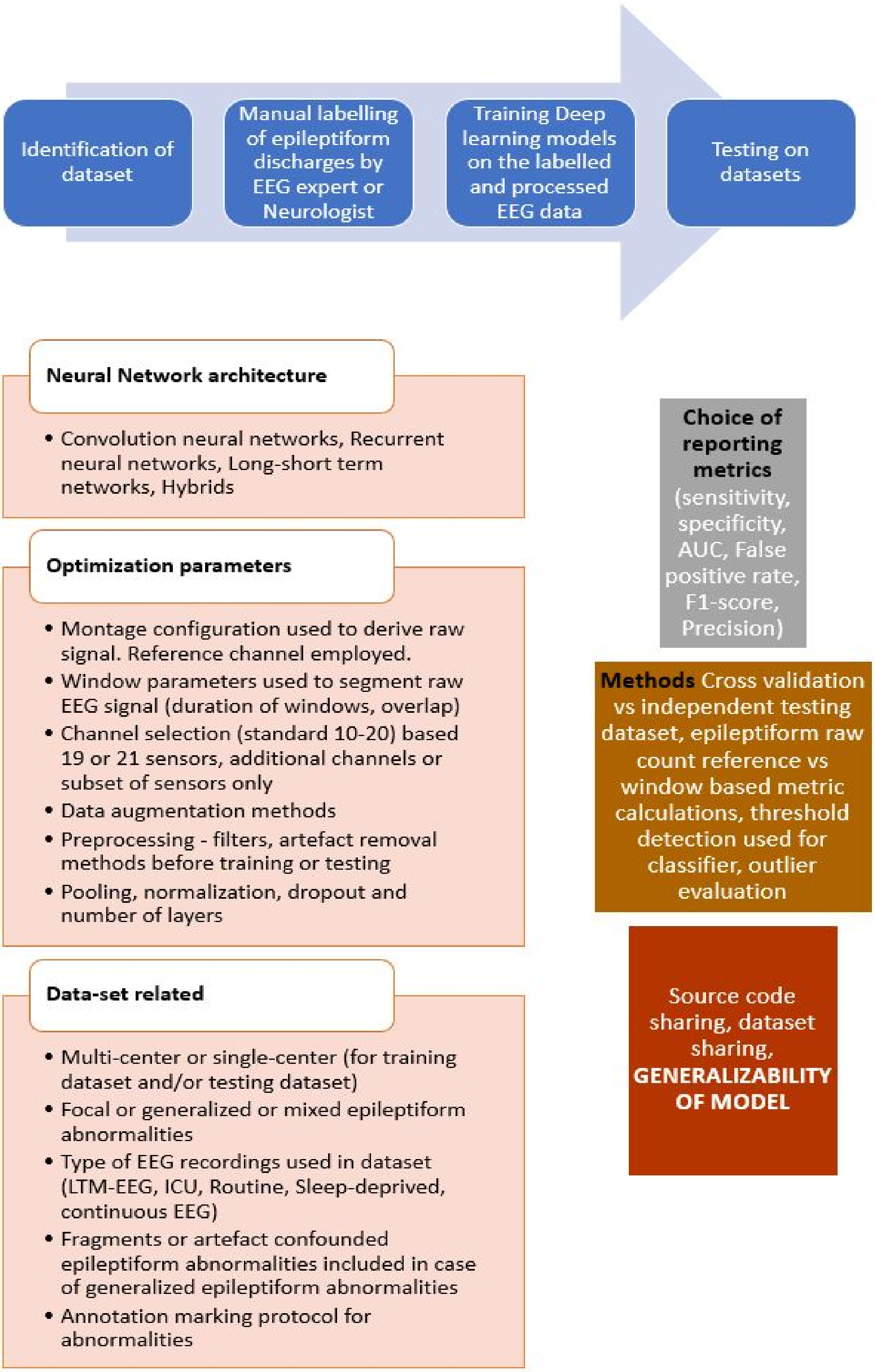


*Figure 4 Deep learning for IED detection framework. Source code and dataset sharing will improve and speed up research in this field.*

Evaluation results are frequently present on test datasets in a summarized pooled manner. A few outlier EEGs causing poor performance may markedly skew the results to show the algorithm as inaccurate whereas this may be the case because of only a few EEGs in which the algorithm failed significantly. In our ongoing work evaluating an unpublished dataset [36]implementing a Graph convolution method (viewing an EEG montage as a graph theory using electrodes as nodes and pair linkage as edges) we found removal of 4 outlier EEGs from a test dataset of 28 EEGs markedly improved precision at a 0.80 detection threshold from 28% to 63% with only a 10% drop in sensitivity (errant spike windows predicted reduced from 781 to 57). Outlier identification efforts, although time-consuming, should therefore be made in conjunction with a trained epileptologist to find the reasons why the overall results of an algorithm may be poor.

Standardized metrics should be reported including as many performance metrics as possible to provide a holistic view rather than a focus on accuracy or AUC. Most importantly and invariably clinical useful metrics of false positive rates and sensitivity should be reported. Reviewers should be looking out for this section carefully as thresholds and selective reporting is possible. The methodology of how the metrics were calculated is important as well. It is important especially that the current priority be that false positive rate be reduced whilst maintaining a high sensitivity.

For Review Only

Cross-testing is vital and will reveal the actual performance of a model. This has only started to be employed in seizure detection. False detections of seizures ranged from 0.15 per hour to 2.5 per hour depending on different datasets used.[64] The hospital and locality ethics of sharing EEG data makes it complex for potential collaborators seeking to implement their model algorithms on external datasets. Multi-centre dataset collation should nevertheless continue to be pursued. Epilepsy centres collaborating will be able to reach target numbers reached in imaging classification by share loading contributions and be able to allow the DL

model to be trained on a large amount of morphologic, topographic and artefactual variation of windows containing epileptiform discharges. This cannot be done without a collaborative mindset.

Source codes detailing current deep learning models being experimented and published in the automated IED literature are not available for other researchers to replicate on their own dataset and subsequently critique, improve or even compare to their own planned models. This very likely may be due to researchers considering that their models could be improved to a point of commercialization or alternatively suggest a lack of confidence on the generalizability of the model and thus keeping model details restricted and neural networks architecture explained in a general way. Such source-code sharing has been done in seizure detection algorithms.[65] Any researcher who has published a model should avail their source code on a public repository to allow people to quickly test and validate the stated model performance on their respective private datasets. This will allow robust peer review. Such feedback can be provided back to the publishing author who can further fine-tune his model or be made aware of the model's performance on different datasets to his. This would be easier than researchers sharing or requesting EEG data from other centres. If source code sharing for cross-testing is not desired the next step would be standardized datasets to be made available in the public domain against which models from different research groups can be tested and compared. This however will not allow peer-review of performance as model testing is carried out by the same authors and selection bias and selective reporting can still result. Thus, an open-source, code-sharing, mindset is definitely required for progress in this field to occur.

A note could be mentioned regarding classifiers being trained for whole EEG in contrast to individual IED marking. Data scientists and research labs interested in this metric should recognize that whole EEG classification will foreseeably remain the domain of human experts due to several reasons. With the advances in ML comes an understanding of the limitations of algorithms and the ethics surrounding their application.[66] Hospital ethics

For Review Only

committees or medical regulatory bodies will unlikely allow computers to make judgements on the labelling of an investigation as normal and abnormal which is to be extended without supervision to clinical care. As a parallel most hospitals and health systems implement automated cardiac telemetry to screen for real-time diagnosis of arrhythmia. Even with the longer history of cardiac telemetry, its less complex signal characteristics and established role, human expertise and oversight is continuously needed so that unnecessary treatment is avoided. Despite this, cases have been reported of invasive interventions based on errant and artefactual automated telemetry results.[67] Governance over automated assessment versus the clinician's assessment of EEG will thus need to be closely monitored for the potential impact on treatment decisions and outcome. The focus instead should be on training, enhancing, and improving the performance of IED classifiers to assist in marking and data reduction with a goal to speeding up the workflow of EEG lab and reviewing staff. It would be unwise to provide improved results on whole EEG classification whilst the underlying goal of improvement desired in hospital practices is IED detection and automated marking.

A great scope of research opportunities present itself in this field. Once a sufficiently accurate or reliable computing model for a validated detection algorithm has been developed, several other opportunities will avail themselves to enhance such models. This could incorporate future work into automated classification of the various abnormal discharges into useful subtypes. This variation can be seen for example in genetic generalized epilepsy or symptomatic generalized epilepsy where several kinds of epileptiform abnormalities can present themselves either between or within a single patient's EEG. The range of heterogeneity of discharges can include typical 2.5 -6 Hz spike/slow wave, fragmented or localized spike or sharps, polyspike trains, polyspike/slow waves, paroxysmal fast activity[68] and also atypical rhythmic or slow spike and wave. Similarly, in focal epilepsy one can get different morphologic, topographic and periodic characteristics including isolated or repetitive runs (brief and long trains) which could be rhythmic or semi-

For Review Only

rhythmic and either confined to a limited topography unilaterally or could be bilateral or multifocal. All this may be further pursued by an upgraded algorithm based on degree of channel involvement via some quantitative criteria. Voltage topographic maps and even more advanced source localization algorithms in high-density EEG could be integrated to easily pre-fill quantitative sections of reports for clinicians. Predictor biomarkers currently being investigated could further allow potential predictability of pharmacoresistance in early clinical stages. Duration of epileptiform discharges and generalized polyspike trains for example are recent quantitative biomarkers associated with drug-resistance.[69,70] Persyst [71] and the Encevis[60]/AIT team have been making progress in some of these domains and commercialized their in-house AI algorithms. However, a systematic study and external validation will be required for more widespread use. This is currently being evaluated by the authors on a multicentre dataset.

# Conclusion

ML and deep learning algorithms, despite success in seizure detection and clinical use, have so far failed to be implemented routinely for epileptiform abnormality detection in clinical care due to inconsistent and uncertain performances. Published algorithms remain doubtful as to their generalizability and are viewed with scepticism when it comes to clinical integration in the real-world setting. Clear protocols need to be devised regarding description of training and testing datasets utilized, annotation methods, IED-benchmarking and more thorough performance evaluation and reporting of metrics. Open sharing of source codes after model publication should be promoted to allow cross-testing and independent validation of algorithms across datasets derived from different research and hospital settings. Despite the current stumbling blocks, a new era in clinical epilepsy diagnostics with automated IED detection is likely to emerge in the near future with deep learning methods at the forefront.

For Review Only

# REFERENCES

1. Gotman J, Gloor P. Automatic Recognition and Quantification of Interictal Epileptic Activity in The Human Scalp EEG. *Electroencephalography and Clinical Neurophysiology*. 1976;41:513-529.

2. Frost JD. Microprocessor-based EEG spike detection and quantification. *International Journal of Bio-Medical Computing*. 1979;10(5):357-373. doi:10.1016/0020-7101(79)90051-5

3. Carrie JRG. A hybrid computer technique for detecting sharp EEG transients. *Electroencephalography and Clinical Neurophysiology*. 1972;33(3):336-338. doi:10.1016/0013-4694(72)90163-0

4. Saini J, Dutta M. An extensive review on development of EEG-based computer-aided diagnosis systems for epilepsy detection. *Network: Computation in Neural Systems*. 2017;28(1):1-27. doi:10.1080/0954898X.2017.1325527

5. Haenssle HA, Fink C, Schneiderbauer R, et al. Man against machine: diagnostic performance of a deep learning convolutional neural network for dermoscopic melanoma recognition in comparison to 58 dermatologists. *Annals of Oncology*. 2018;29(8):1836-1842. doi:10.1093/annonc/mdy166

6. Gulshan V, Peng L, Coram M, et al. Development and Validation of a Deep Learning Algorithm for Detection of Diabetic Retinopathy in Retinal Fundus Photographs. *JAMA*. 2016;316(22):2402. doi:10.1001/jama.2016.17216

7. Beede E, Baylor E, Hersch F, et al. A Human-Centered Evaluation of a Deep Learning System Deployed in Clinics for the Detection of Diabetic Retinopathy. In: *Proceedings of the 2020 CHI Conference on Human Factors in Computing Systems*. ACM; 2020:1-12. doi:10.1145/3313831.3376718

8. Roy Y, Banville H, Albuquerque I, Gramfort A, Falk TH, Faubert J. Deep learning-based electroencephalography analysis: a systematic review. *J Neural Eng*. 2019;16(5):051001. doi:10.1088/1741-2552/ab260c

9. Craik A, He Y, Contreras-Vidal JL. Deep learning for electroencephalogram (EEG) classification tasks: a review. *J Neural Eng*. 2019;16(3):031001. doi:10.1088/1741-2552/ab0ab5

10. Kamitaki BK, Yum A, Lee J, et al. Yield of conventional and automated seizure detection methods in the epilepsy monitoring unit. *Seizure*. 2019;69:290-295. doi:10.1016/j.seizure.2019.05.019

11. Reus EEM, Visser GH, Cox FME. Using sampled visual EEG review in combination with automated detection software at the EMU. *Seizure - European Journal of Epilepsy*. 2020;80:96-99. doi:10.1016/j.seizure.2020.06.002

For Review Only

12. Rasheed K, Qayyum A, Qadir J, et al. Machine Learning for Predicting Epileptic Seizures Using EEG Signals: A Review. *arXiv:200201925 [cs, eess, q-bio]*. Published online February 4, 2020. Accessed July 23, 2021. http://arxiv.org/abs/2002.01925

13. da Silva Lourenço C, Tjepkema-Cloostermans MC, van Putten MJAM. Efficient use of clinical EEG data for deep learning in epilepsy. *Clinical Neurophysiology*. 2021;132(6):1234-1240. doi:10.1016/j.clinph.2021.01.035

14. de Jong J, Cutcutache I, Page M, et al. Towards realizing the vision of precision medicine: AI based prediction of clinical drug response. *Brain*. 2021;144(6):1738-1750. doi:10.1093/brain/awab108

15. Abbasi B, Goldenholz DM. Machine learning applications in epilepsy. *Epilepsia*. 2019;60(10):2037-2047. doi:10.1111/epi.16333

16. Jing J, Sun H, Kim JA, et al. Development of Expert-Level Automated Detection of Epileptiform Discharges During Electroencephalogram Interpretation. *JAMA Neurol*. Published online October 21, 2019. doi:10.1001/jamaneurol.2019.3485

17. Brogger J, Eichele T, Aanestad E, Olberg H, Hjelland I, Aurlien H. Visual EEG reviewing times with SCORE EEG. *Clinical Neurophysiology Practice*. 2018;3:59-64. doi:10.1016/j.cnp.2018.03.002

18. Moura LMVR, Shafi MM, Ng M, et al. Spectrogram screening of adult EEGs is sensitive and efficient. *Neurology*. 2014;83(1):56-64. doi:10.1212/WNL.0000000000000537

19. Wei B, Zhao X, Shi L, Xu L, Liu T, Zhang J. A deep learning framework with multi-perspective fusion for interictal epileptiform discharges detection in scalp electroencephalogram. *J Neural Eng*. 2021;18(4):0460b3. doi:10.1088/1741-2552/ac0d60

20. Tatum WO. How not to read an EEG: Introductory statements. *Neurology*. 2013;80(Issue 1, Supplement 1):S1-S3. doi:10.1212/WNL.0b013e318279730e

21. Rathore C, Prakash S, Rana K, Makwana P. Prevalence of benign epileptiform variants from an EEG laboratory in India and frequency of their misinterpretation. *Epilepsy Research*. 2021;170:106539. doi:10.1016/j.eplepsyres.2020.106539

22. Benbadis SR, Kaplan PW. The Dangers of Over-Reading an EEG: *Journal of Clinical Neurophysiology*. 2019;36(4):249. doi:10.1097/WNP.0000000000000598

23. Tatum WO, Selioutski O, Ochoa JG, et al. American Clinical Neurophysiology Society Guideline 7: Guidelines for EEG Reporting. *The Neurodiagnostic Journal*. 2016;56(4):285-293. doi:10.1080/21646821.2016.1245576

24. Moore JL, Carvalho DZ, St Louis EK, Bazil C. Sleep and Epilepsy: a Focused Review of Pathophysiology, Clinical Syndromes, Co-morbidities, and Therapy. *Neurotherapeutics*. 2021;18(1):170-180. doi:10.1007/s13311-021-01021-w

25. Grigg-Damberger M, Foldvary-Schaefer N. Bidirectional relationships of sleep and epilepsy in adults with epilepsy. *Epilepsy & Behavior*. 2021;116:107735. doi:10.1016/j.yebeh.2020.107735

26. Drake ME, Pakalnis A, Phillips BB, Denio LS. Sleep and sleep deprived EEG in partial and generalized epilepsy. *Acta Neurol Belg*. 1990;90(1):11-19.


For Review Only

27. Seneviratne U, Lai A, Cook M, D'Souza W, Boston RC. "Sleep Surge": The impact of sleep onset and offset on epileptiform discharges in idiopathic generalized epilepsies. *Clinical Neurophysiology*. 2020;131(5):1044-1050. doi:10.1016/j.clinph.2020.01.021

28. Dash D, Hernandez-Ronquillo L, Moien-Afshari F, Tellez-Zenteno JF. Ambulatory EEG: a cost-effective alternative to inpatient video-EEG in adult patients. *Epileptic Disorders*. 2012;14(3):290-297. doi:10.1684/epd.2012.0529

29. Seneviratne U, D'Souza WJ. Chapter 10 - Ambulatory EEG. In: Levin KH, Chauvel P, eds. *Handbook of Clinical Neurology*. Vol 160. Clinical Neurophysiology: Basis and Technical Aspects. Elsevier; 2019:161-170. doi:10.1016/B978-0-444-64032-1.00010-2

30. Young GB, Mantia J. Continuous EEG monitoring in the intensive care unit. In: *Handbook of Clinical Neurology*. Vol 140. Elsevier; 2017:107-116. doi:10.1016/B978-0-444-63600-3.00007-6

31. Louis EKS, Frey LC, Britton JW, et al. *The Normal EEG*. American Epilepsy Society; 2016. Accessed July 8, 2021. https://www.ncbi.nlm.nih.gov/books/NBK390343/

32. McKay JH, Tatum WO. Artifact Mimicking Ictal Epileptiform Activity in EEG. [Review]. *Journal of Clinical Neurophysiology*. 2019;36(4):275-288. doi:10.1097/WNP.0000000000000597

33. Mari-Acevedo J, Yelvington K, Tatum WO. Normal EEG variants. *Handb Clin Neurol*. 2019;160:143-160. doi:10.1016/B978-0-444-64032-1.00009-6

34. Kang JY, Krauss GL. Normal Variants Are Commonly Overread as Interictal Epileptiform Abnormalities. [Review]. *Journal of Clinical Neurophysiology*. 2019;36(4):257-263. doi:10.1097/WNP.0000000000000613

35. Nayak CS, Anilkumar AC. EEG Normal Sleep. In: *StatPearls*. StatPearls Publishing; 2021. Accessed July 21, 2021. http://www.ncbi.nlm.nih.gov/books/NBK537023/

36. D. Nhu LK. Graph Convolutional Network For Generalized Epileptiform Abnormality Detection On EEG. *IEEE Signal Process Med Biol Symp SPMB*. 2021;In Press.

37. Lin L, Drislane FW. Lateralized Periodic Discharges: A Literature Review. *Journal of Clinical Neurophysiology*. 2018;35(3):189-198. doi:10.1097/WNP.0000000000000448

38. Meritam Larsen P, Wüstenhagen S, Terney D, et al. Photoparoxysmal response and its characteristics in a large EEG database using the SCORE system. *Clin Neurophysiol*. 2021;132(2):365-371. doi:10.1016/j.clinph.2020.10.029

39. Buluş E, Abanoz Y, Gülen Abanoz Y, Yeni SN. The Effect of Cognitive Tasks During Electroencephalography Recording in Patients With Reflex Seizures. *Clin EEG Neurosci*. Published online December 23, 2020:1550059420983622. doi:10.1177/1550059420983622

40. Gelžinienė G, Endzinienė M, Jurkevičienė G. EEG activation by neuropsychological tasks in idiopathic generalized epilepsy of adolescence. *Brain Dev*. 2015;37(4):409-417. doi:10.1016/j.braindev.2014.06.013

41. Webber WRS, Litt B, Lesser RP, Fisher RS, Bankman I. Automatic EEG spike detection: what should the computer imitate? *Electroencephalography and Clinical Neurophysiology*. 1993;87(6):364-373. doi:10.1016/0013-4694(93)90149-P


For Review Only

42. Wilson SB, Harner RN, Duffy FH, Tharp BR, Nuwer MR, Sperling MR. Spike detection. I. Correlation and reliability of human experts. *Electroencephalography and Clinical Neurophysiology*. 1996;98(3):186-198. doi:10.1016/0013-4694(95)00221-9

43. Bagheri E, Dauwels J, Dean BC, Waters CG, Westover MB, Halford JJ. Interictal epileptiform discharge characteristics underlying expert interrater agreement. *Clin Neurophysiol*. 2017;128(10):1994-2005. doi:10.1016/j.clinph.2017.06.252

44. Kural MA, Duez L, Sejer Hansen V, et al. Criteria for defining interictal epileptiform discharges in EEG: A clinical validation study. *Neurology*. 2020;94(20):e2139-e2147. doi:10.1212/WNL.0000000000009439

45. Beniczky S, Aurlien H, Franceschetti S, et al. Interrater agreement of classification of photoparoxysmal electroencephalographic response. *Epilepsia*. 2020;61(9):e124-e128. doi:10.1111/epi.16655

46. Piccinelli P, Viri M, Zucca C, et al. Inter-rater reliability of the EEG reading in patients with childhood idiopathic epilepsy. *Epilepsy Research*. 2005;66(1):195-198. doi:10.1016/j.eplepsyres.2005.07.004

47. Halford JJ, Arain A, Kalamangalam GP, et al. Characteristics of EEG Interpreters Associated With Higher Interrater Agreement. *J Clin Neurophysiol*. 2017;34(2):168-173. doi:10.1097/WNP.0000000000000344

48. Golmohammadi M, Harati Nejad Torbati AH, Lopez de Diego S, Obeid I, Picone J. Automatic Analysis of EEGs Using Big Data and Hybrid Deep Learning Architectures. *Front Hum Neurosci*. 2019;13. doi:10.3389/fnhum.2019.00076

49. Seneviratne U, Hepworth G, Cook M, D'Souza W. Atypical EEG abnormalities in genetic generalized epilepsies. *Clinical Neurophysiology*. 2016;127(1):214-220. doi:10.1016/j.clinph.2015.05.031

50. Obeid I, Picone J. The Temple University Hospital EEG Data Corpus. *Front Neurosci*. 2016;10. doi:10.3389/fnins.2016.00196

51. Lourenço C, Tjepkema-Cloostermans MC, Teixeira LF, van Putten MJAM. Deep Learning for Interictal Epileptiform Discharge Detection from Scalp EEG Recordings. In: *IFMBE Proceedings*. Vol 76. ; 2020:1984-1997. doi:10.1007/978-3-030-31635-8_237

52. Prasanth T, Thomas J, Yuvaraj R, et al. Deep Learning for Interictal Epileptiform Spike Detection from scalp EEG frequency sub bands. *Annu Int Conf IEEE Eng Med Biol Soc*. 2020;2020:3703-3706. doi:10.1109/EMBC44109.2020.9175644

53. Thomas J, Thangavel P, Peh WY, et al. Automated Adult Epilepsy Diagnostic Tool Based on Interictal Scalp Electroencephalogram Characteristics: A Six-Center Study. *Int J Neural Syst*. Published online January 12, 2021:2050074. doi:10.1142/S0129065720500744

54. Fukumori K. Fully Data-driven Convolutional Filters with Deep Learning Models for Epileptic Spike Detection. *ICASSP, IEEE International Conference on Acoustics, Speech and Signal Processing - Proceedings*. 2019;2019:2772-2776. doi:10.1109/ICASSP.2019.8682196

55. Thomas J, Comoretto L, Jin J, Dauwels J, Cash SS, Westover MB. EEG CLassification Via Convolutional Neural Network-Based Interictal Epileptiform Event Detection. In:

For Review Only

*2018 40th Annual International Conference of the IEEE Engineering in Medicine and Biology Society (EMBC)*. ; 2018:3148-3151. doi:10.1109/EMBC.2018.8512930

56. Xu Z, Wang T, Cao J, Bao Z, Jiang T, Gao F. BECT Spike Detection Based on Novel EEG Sequence Features and LSTM Algorithms. *IEEE Trans Neural Syst Rehabil Eng*. 2021;29:1734-1743. doi:10.1109/TNSRE.2021.3107142

57. Lashgari E, Liang D, Maoz U. Data augmentation for deep-learning-based electroencephalography. *J Neurosci Methods*. 2020;346:108885. doi:10.1016/j.jneumeth.2020.108885

58. Koren J, Hafner S, Feigl M, Baumgartner C. Systematic analysis and comparison of commercial seizure-detection software. *Epilepsia*. 2021;62(2):426-438. doi:10.1111/epi.16812

59. Jaramillo M. Persyst: The worldwide leader in EEG software. Persyst. Accessed October 8, 2021. https://www.persyst.com/

60. Spike detection - Encevis. encevis. Accessed October 3, 2021. https://www.encevis.com/solutions/spike-detection/

61. Fürbass F, Kural MA, Gritsch G, Hartmann M, Kluge T, Beniczky S. An artificial intelligence-based EEG algorithm for detection of epileptiform EEG discharges: Validation against the diagnostic gold standard. *Clinical Neurophysiology*. 2020;131(6):1174-1179. doi:10.1016/j.clinph.2020.02.032

62. Scheuer M. Spike detection: Inter-reader agreement and a statistical Turing test on a large data set. *Clinical Neurophysiology*. 2017;128(1):243-250. doi:10.1016/j.clinph.2016.11.005

63. Halford JJ, Westover MB, LaRoche SM, et al. Interictal Epileptiform Discharge Detection in EEG in Different Practice Settings: *Journal of Clinical Neurophysiology*. 2018;35(5):375-380. doi:10.1097/WNP.0000000000000492

64. Raghu S, Sriraam N, Gommer ED, et al. Cross-database evaluation of EEG based epileptic seizures detection driven by adaptive median feature baseline correction. *Clinical Neurophysiology*. 2020;131(7):1567-1578. doi:10.1016/j.clinph.2020.03.033

65. Bernabei JM, Owoputi O, Small SD, et al. A Full-Stack Application for Detecting Seizures and Reducing Data During Continuous Electroencephalogram Monitoring. *Crit Care Explor*. 2021;3(7):e0476. doi:10.1097/CCE.0000000000000476

66. Grote T, Berens P. On the ethics of algorithmic decision-making in healthcare. *J Med Ethics*. 2020;46(3):205-211. doi:10.1136/medethics-2019-105586

67. Henriques-Forsythe MN, Ivonye CC, Jamched U, Kamuguisha LKK, Olejeme KA, Onwuanyi AE. Is telemetry overused? Is it as helpful as thought? *CCJM*. 2009;76(6):368-372. doi:10.3949/ccjm.76a.07260

68. Sagi V, Kim I, Bhatt AB, Sonmezturk H, Abou-Khalil BW, Arain AM. Generalized paroxysmal fast activity in EEG: An unrecognized finding in genetic generalized epilepsy. *Epilepsy & Behavior*. 2017;76:101-104. doi:10.1016/j.yebeh.2017.08.019

For Review Only

69. Sun Y, Seneviratne U, Perucca P, et al. Generalized polyspike train: An EEG biomarker of drug-resistant idiopathic generalized epilepsy. *Neurology*. 2018;91(19):e1822-e1830. doi:10.1212/WNL.0000000000006472

70. Arntsen V, Sand T, Syvertsen MR, Brodtkorb E. Prolonged epileptiform EEG runs are associated with persistent seizures in juvenile myoclonic epilepsy. *Epilepsy Research*. 2017;134:26-32. doi:10.1016/j.eplepsyres.2017.05.003

71. Spike Detection - Persyst. Persyst. Accessed October 3, 2021. https://www.persyst.com/technology/spike-detection/

# Authors contributions

Mubeen Janmohamed performed literature search and was primary draft author, format design and editor of manuscript. Duong Nhu was second author and performed literature search, editing, formatting and review of manuscript. Levin Kuhlman, Amanda Gilligan, Chang Wei Tan, Piero Perucca, Terence J. O'Brien edited and reviewed the manuscript. Patrick Kwan was the primary supervisor, editor and review of article.

# Role of the funding source

Mubeen Janmohamed receives support through an "Australian Government Research Training Program (RTP) Scholarship" for PhD at Monash University, Central Clinical School, Melbourne Australia. Patrick Kwan is supported by the Medical Research Future Fund Practitioner Fellowship (MRF1136427). Levin Kuhlmann is supported by the National Health and Medical Research Council (GNT1183119; GNT1160815) and the Epilepsy Foundation of America. Duong Nhu is supported by the Graduate Research Industry Scholarship (GRIP) at Monash University, Australia. Piero Perucca is supported by the National Health and Medical Research Council (APP1163708), the Epilepsy Foundation, The University of Melbourne, Monash University, Brain Australia, and the Weary Dunlop Medical Research

For Review Only

Foundation. Terence O'Brien was supported by by a NHMRC Investigator Grant (APP1176426).

# Disclosure of Conflicts of Interest

Outside the submitted work, PP has received speaker honoraria or consultancy fees to his institution from Chiesi, Eisai, LivaNova, Novartis, Sun Pharma, Supernus, and UCB Pharma. He is an Associate Editor for Epilepsia Open. PK's institution has received research grants from Biscayne Pharmaceuticals, Eisai, GW Pharmaceuticals, LivaNova, Novartis, UCB Pharma, and Zynerba outside the submitted work; he has received speaker fees from Eisai, LivaNova, and UCB Pharma, outside the submitted work. PK's institution has received research grants from Biscayne Pharmaceuticals, Eisai, GW Pharmaceuticals, LivaNova, Novartis, UCB Pharma, and Zynerba outside the submitted work; he has received speaker fees from Eisai, LivaNova, and UCB Pharma, outside the submitted work.

For Review Only

## List of figures

**Figure 1** The structure of data available from hospital-based EEG servers

**Figure 2** Epileptiform variation in GGE EEG datasets (From Left to right)

**Figure 3** Artefacts mimicking Inter-ictal epileptiform abnormalities

**Figure 4** Deep learning for IED detection framework. Source code and dataset sharing will improve and speed up research in this field.

## List of Tables

**Table 1** Pros and Cons of future computer-assisted detection in EEG laboratories

**Table 2:** Performance metrics commonly used in machine learning studies

For Review Only

**Table 1** *Pros and Cons of future computer-assisted detection in EEG laboratories*

## Pros

- Super-speed labelling and substantial data reduction leading to faster workflows
- Substituting unavailable expertise in low-resource countries
- Artificial intelligence is purported to have the potential of better results than traditionally trained experts.

## Cons

- Missed true epileptiform discharges (false negatives).
- Exaggerated labelling of artefacts as abnormalities (false positives)
- Reduction of job and learning opportunities for EEG scientists and epilepsy trainees.

For Review Only

**Table 2:** *Performance metrics commonly used in machine learning studies*

## Metrics of clinical utility for IED detection

- **Sensitivity**: Proportion of true gold standard IEDs correctly detected
- **Precision:** The proportion of true marked gold standard IEDs to all machine predicted positive labels. (True Positives)/(True positives + False positives)
- **False positive rate:** Rate of false positives which were not classified by the gold standard as IEDs typically reported in per hour.
- **F1-score –** This takes into account the two most relevant metrics of precision and recall.
- **AUPRC** - Area under the precision-recall curve (AUPRC) which differs from the area under the ROC curve. A model achieves perfect score when it identifies all epileptiform abnormalities without marking normal or benign abnormalities.

## Metrics of limited clinical utility in isolation

- True negatives, specificity, accuracy and AUROC (area under ROC curve).

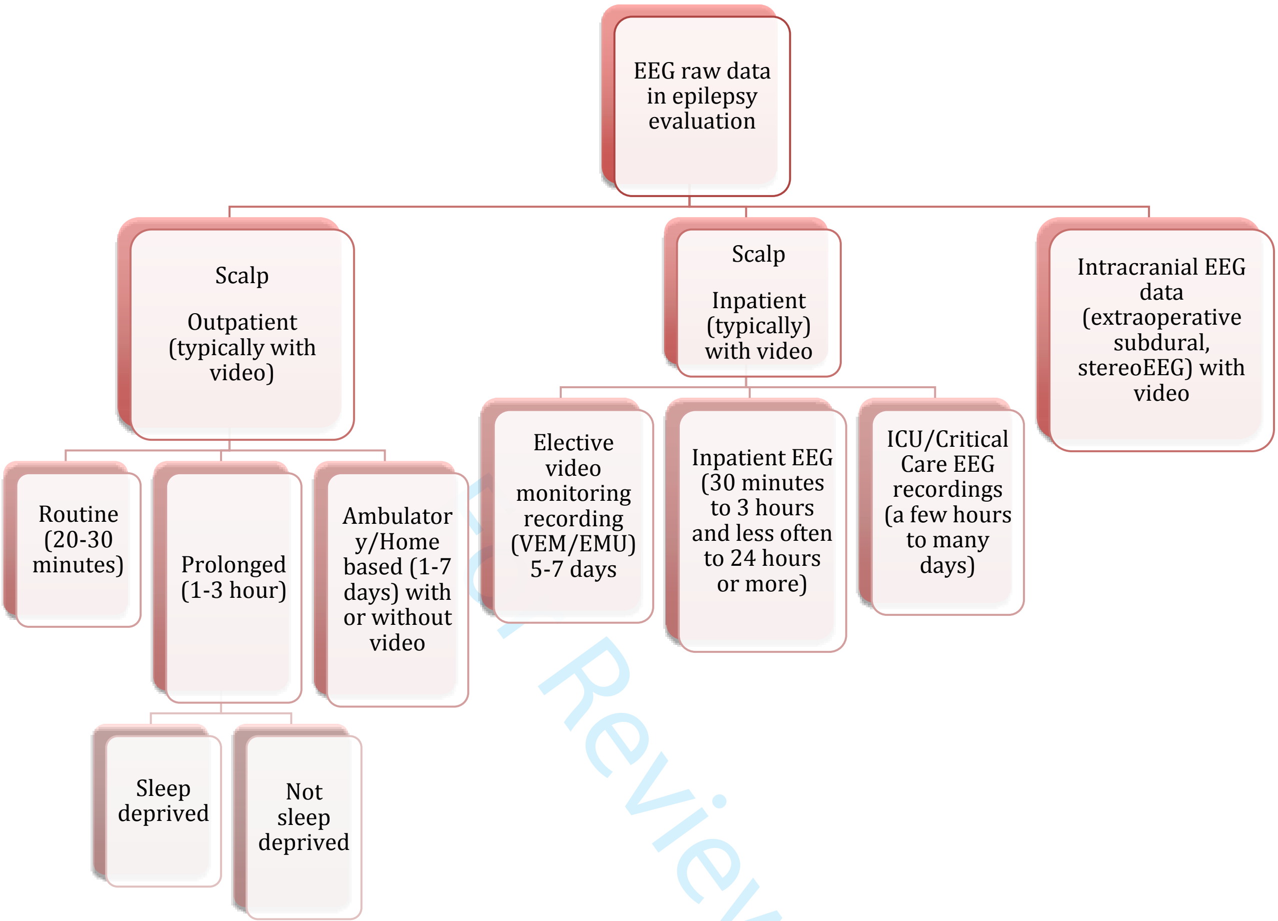


*Figure 1 The structure of data available from hospital-based EEG servers*

For Review Only

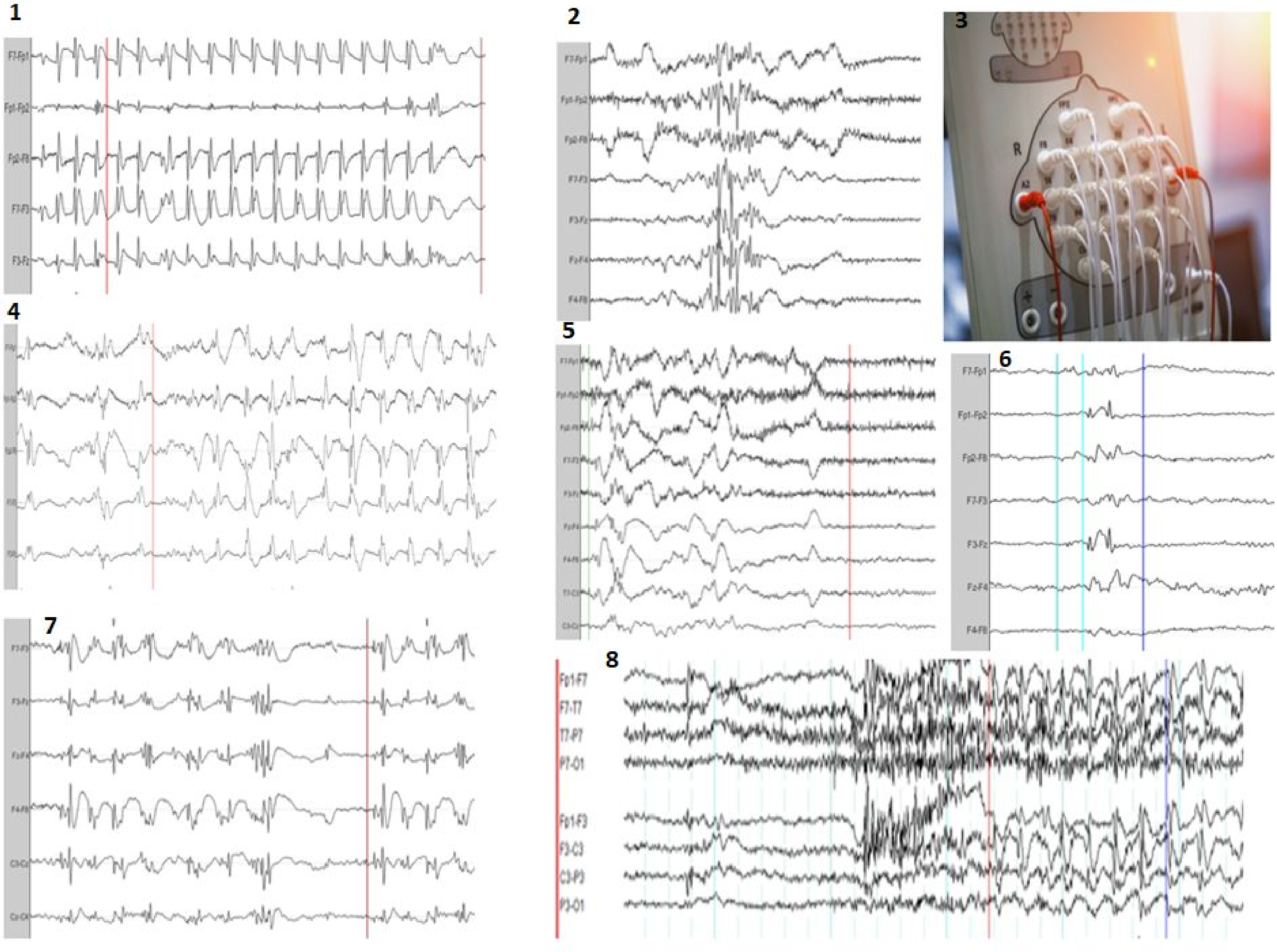


*Figure 2 Epileptiform variation in GGE EEG datasets (From Left to right) 1. Classic 3 Hz spike and wave on transverse montage, 2. polyspikes with EMG artefact in frontopolar channels and eye movements, 3. EEG amplifier for 10-20 recording, 4. slow spike/wave on transverse montage, 5. mild EMG affecting frontal channels with embedded small spike and waves and irregular slow waves, 6. fragments on transverse montage, 7.polyspike/slow waves on transverse montage, 8. marked EMG artefact confounding epileptiform abnormality in temporal and frontal channels on longitudinal montage*

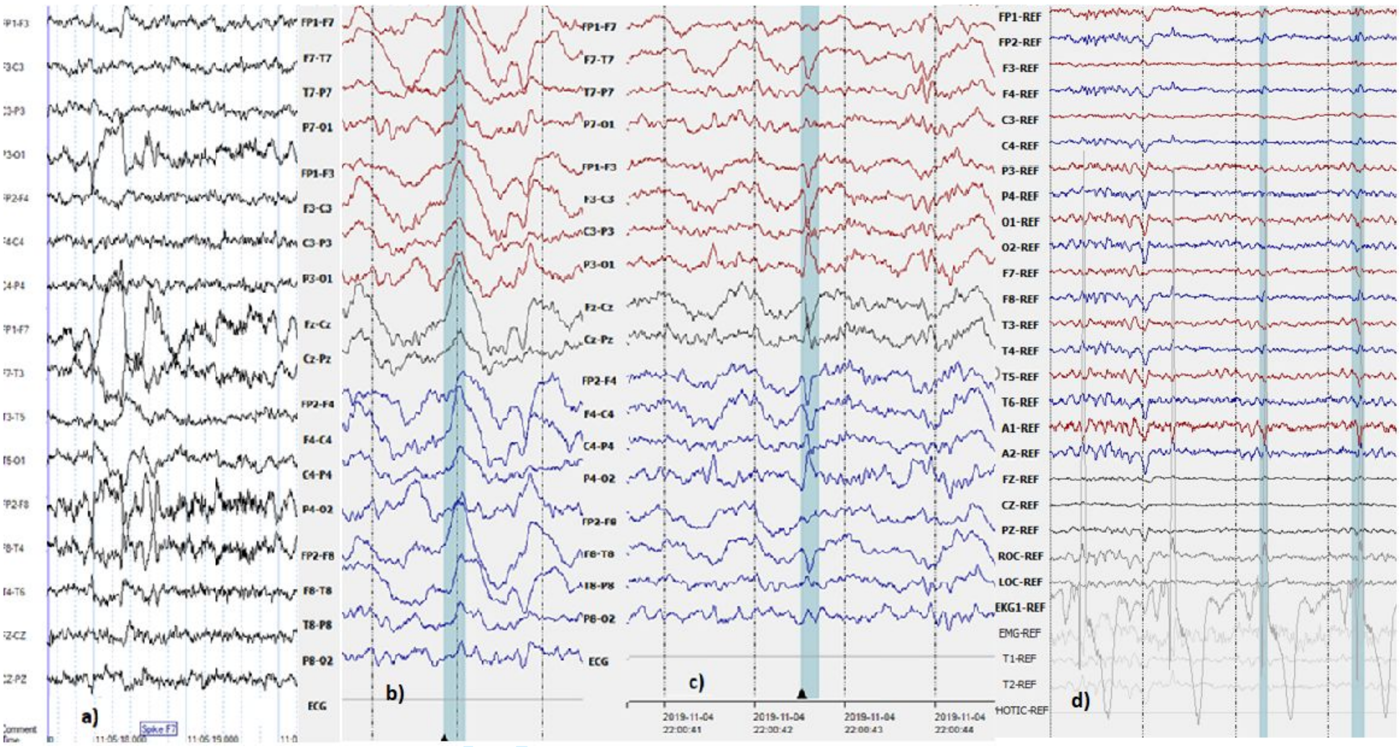


*Figure 3 IED mimics a) Eye and EMG resembling IED mimic detected by algorithm. b) high amplitude slow wave in Stage 3 sleep causing false positive c) v-wave mimicking sharp wave and labelled as abnormal by algorithm d) ECG artefact picked up as train of IEDs*

For Review Only

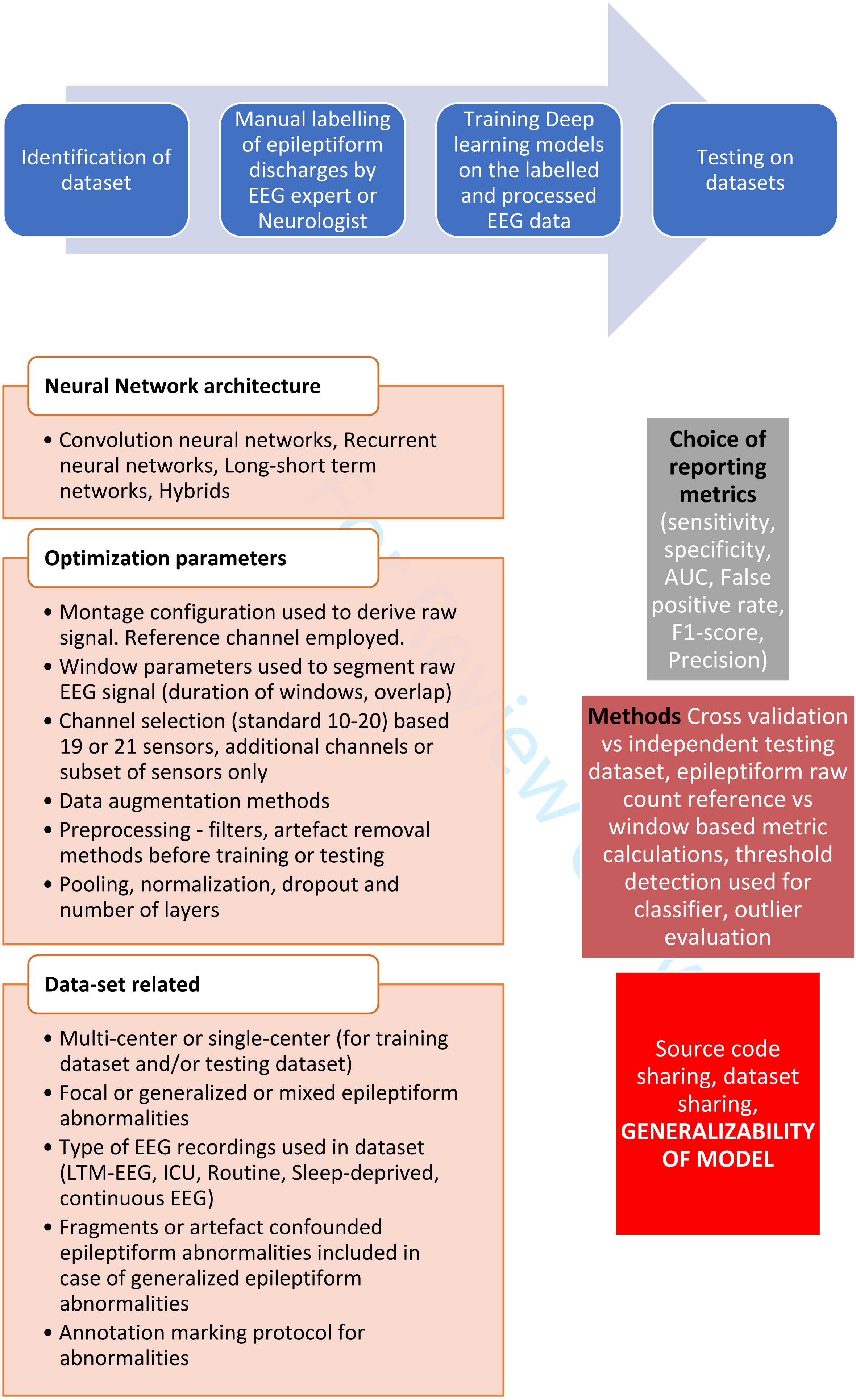


*Figure 3 Deep learning for IED detection framework. Source code and dataset sharing will improve and speed up research in this field.*
