# Supplementary material for: Moving the field forward: detection of epileptiform abnormalities on scalp electroencephalography using deep learning—clinical application perspectives
Source: Brain Commun. 2022 Aug 29;4(5):fcac218. doi: 10.1093/braincomms/fcac218 (PMC9453433; doi:10.1093/braincomms/fcac218)
Supplement: fcac218_Supplementary_Data [file Original_submission_decision_letter.pdf › Original_submission_decision_letter.pdf]

From: brcoms.editorialoffice@oup.com

To: mubeen.janmohamed@monash.edu

CC:

BCC:

Subject: Brain Communications - Decision on Manuscript ID BRAINCOM-2021-396

Body: 01-Apr-2022

Dear Dr. Janmohamed,

Thank you for your recent submission BRAINCOM-2021-396 entitled "Moving the field forward: detection of epileptiform abnormalities on scalp electroencephalography using deep learning.
Clinical application perspectives" to Brain Communications.

Your manuscript has been reviewed, and while the reviewers and editorial team appreciate many aspects of the paper, they raise some concerns that you will need to address in order for the paper to be considered for publication.

If you can address these concerns, I invite you to submit a revised manuscript and a response to each of the reviewers' comments. The reviewer(s)' comments are included at the bottom of this message.

In addition to the reviewers' comments, please also address our Scientific Editor's comments added below to comply with our requirements for presentation of scientific data at Brain Communications.

Please format your revised manuscript according to our Instructions to Authors https://academic.oup.com/braincomms/pages/General_Instructions – a summary of key points is attached to help with this.

To revise your manuscript, click the link below or log into https://mc.manuscriptcentral.com/braincom and enter your Author Centre, where you will find your manuscript title listed under "Manuscripts with Decisions." Under "Actions," click on "Create a Revision." Your manuscript number will be appended to denote a revision.

*** PLEASE NOTE: This is a two-step process. After clicking on the link, you will be directed to a webpage to confirm. ***

https://mc.manuscriptcentral.com/braincom?URL_MASK=59db0c1a7f044a27a5f26f59cf691dcb

Please upload a revised manuscript with changes tracked with track changes or in coloured text.

When submitting your revised manuscript, you will be asked to respond to the

reviewer(s) comments. Please be as specific as possible in your response to all of the comments.

The editorial team at Brain Communications aims to increase transparency in scientific publishing and to demonstrate the value that peer review brings to the scientific process. Please therefore consider whether you are willing to have reviewer(s)' comments on your paper and your response published as supplementary data with the paper if it is accepted. This is entirely optional and will only be done if both the authors and reviewers agree. While submitting your revision there will be an opportunity to review or update the answer you gave previously, if necessary.

IMPORTANT: Your original files are available to you when you upload your revised manuscript. Please delete any redundant files before completing the submission.

Because we are trying to facilitate timely publication of manuscripts submitted to Brain Communications, we would appreciate your revised manuscript as soon as possible. If it is not possible for you to resubmit within 3 months, the option to submit a revision will expire, and we would then consider your revised paper as a new submission. However, if you need a little more time, please get in touch and we can extend this deadline for you.

Once again, thank you for submitting your manuscript to Brain Communications and I look forward to receiving your revision.

Best wishes

Prof. Richard Chin
Associate Editor, Brain Communications

Professor Tara Spires-Jones
Founding Editor, Brain Communications

Reviewer(s)' Comments to Author:

Reviewer: 1 Michel van Putten

Comments to the Author
Moving the field forward: detection of epileptiform abnormalities on scalp electroencephalography using deep learning.

This article aims to review the applications of deep learning to automate the detection of "epileptiform abnormalities". However, the main focus of the work is on the type of data and challenges the field of automated epileptiform abnormality detection faces, rather than on the methods that have been applied so far and the results they obtained.

A clear flow of information is lacking, and the goal of the review appears to change throughout the work (that is too lengthy, as well). Text oftentimes contains trivialities, including textbook knowledge on sensitivity/specificity/precision and more.. one may assume this is known by the readers of this journal. Further, most illustrations must be improved, as well, and sometimes contain trivial details, too (“an EEG amplifier”). Detailed comments below (but are non-exhaustive)

MAJOR COMMENTS
-In the first paragraph of the introduction, the authors correctly distinguish machine learning (ML) form deep learning (DL). However, in the following paragraphs and throughout the article, ML is several times used to refer to DL approaches, suggesting it is a synonym

-In the introduction, one article employing DL methods is mentioned in detail without a convincing explanation of why it was chosen in detriment of other articles.

-In the last paragraph of the introduction, this work is once more described as a ‘review’ that ‘summarizes some of the perspectives of clinicians’. It is not clean to which extent that is actually a review.

-The name of the first section, ‘Overview of scalp EEG datasets available for machine learning’ is once again inconsistent with the supposed DL focus of this work.

-The structure of the article is not logical: it goes from EEG data to performance testing, back to data annotation and then metrics evaluation, which is in turned followed by ‘How should an IED model fit for clinical integration be reported – focus on the metrics’ and then ‘Whole EEG Classification’. There is no cohesive storyline throughout the article.

-In line 60 of page 6 / line 3 of page 7, one can read ‘Amongst 101 subjects whose previous raw EEGs were accessible, 30% were noted to be misinterpreted as epileptiform abnormalities.’. It is not clear what ‘misinterpreted as epileptiform abnormalities’ means.

-The sentences in lines 3-10 of page 8 are unclear, and referring to a type of data as ‘a common dataset’ is scientifically incorrect.

-The sentences in lines 50-58 of page 10 have no connection to the following paragraph, so it is not clear why they are placed in the beginning of that section.

-Figure 2 is composed of generalized EEG epochs, artefacts and other sub-images. The purpose of this figure is not clear, or why generalized epilepsy was chosen to represent pattern variability.

-Figure 3 is composed of random EEG epochs that were supposedly misdetected by DL algorithms. However, it is not said by which algorithm, or how it was trained or with what type of data. Furthermore, it is only referenced once in the main text, between brackets, without any further explanation of its relevance.

-The first sentence of section ‘Proposed Hierarchy for gold standard epileptiform detection’ is poorly written and the concept of ‘hierarchical order’ in this context is not explained to a satisfactory extent.

-Despite barely mentioning specific articles that use deep learning methods throughout this work, the authors refer to their own ‘ongoing work evaluating an unpublished dataset’ to support an argument. This does not make sense in such a review paper.

- Metrics evaluation (page 18): this part is not clear, and authors should better distinguish between the performance of a particular algorithm and the relation to the priors. For instance, accuracy is a function of both the ‘test quality’ (i.e. the senstivity and specificity) AND the prior likelihood (with one exceptation in the unlikely circumstance that sensitivity and specifiticy are equal). Accuracy is therefore NOT the most intuitive measure to ‘understand’ (line 24) if different algorithms are compared (as typically priors are different).

- Authors suggest this to be a review, and they should report to which extent the various performance metrics have actually been reported in the literature. There is not even an overview of the papers studied, what epilepsy types were included, if an independent set for evaluation was used, the size and type of the EEGs used, etc.

Minor comments
-In the abstract, the word ‘discharges’ is missing in the expression ‘inter-ictal epileptiform discharges (IEDs)’.
-The citation style is not uniform (eg. lines 41/42 of page 13).
-Lines 56-60 of page 14, lines 8-13 of page 15 and lines 52-54 of page 16 need citations.

Reviewer: 2 Umesh Vivekananda

Comments to the Author
In this review Janmohamed et al review the existing approaches to automated interictal epileptiform abnormality detection, in particular the advent of machine (+deep) learning. They discuss the rationale, limitations, and evaluation of ML approaches. This is a broad and thoughtful piece that will inform the broad neurological community of such technology. The demand raised by the authors for open access to source code is welcome. Some minor points to consider are
1. Less is mentioned about how ML deals with the data (e.g. multivariate pattern analysis, independent component analysis, estimators, predictors) and more on the statistical output (such as AUC, specificity). So some information on common ML approaches might be helpful.
2. A table of the biggest ML studies in IED detection may be a good point of reference for readers
3. One of the interesting future applications of such technology might be within the wearable realm and real time analysis of EEG data for the patient in home studies
4. Although consideration of montage of the data is mentioned, it would be useful

to know if a particular montage is adopted for ML in the existing literature
5. Within the metric section one promising field that might be mentioned is a Bayesian approach to analysis (https://www.infona.pl/resource/bwmeta1.element.elsevier-b7bd05ba-b5ab-33bc-9054-086ec7163c6f)
6. Small point but when mentioning single channel evaluation (p21) there is the limitation of not appreciating the field associated with an IED, that would require neighbouring channel data
7. Also when mentioning time windows in p22 a small description of the difference between contiguous, sliding windows might be helpful
8. Although likely outside the scope of this review, would be a ML approach to pathogenic high frequency oscillations

Comments from the Scientific Editor:

-Please supply a graphical abstract. A graphical abstract is a visual representation of the central finding or methodology of the paper. It will be displayed in the table of contents. Authors should strive to make the graphical abstract informative, interesting, visually appealing, and straightforward (no caption needed, no abbreviations except gene names). Please upload as a separate high resolution image file (tiff preferred). The technical requirements for the graphical abstracts are:
  --size of the submitted image: 1200 pixels square at 300dpi
  --font of the text: Arial 12-16 point (smaller fonts will not be legible online)

-Please add an Abbreviated Summary (up to 50 words) of your paper that captures the main purpose and conclusions of the work. The summary should state the main results written in the third person (‘[1st author surname] et al. report that …’) and conclusion. This summary will be used in the contents list online and should not contain any abbreviations other than those on the accepted abbreviations list and accepted gene/protein names, making it accessible to the non-specialist. Please upload it as a Word document. Allowed abbreviations: AIDS; ANOVA; AMPA; ATP; A,T,C,G; CNS; CSF; CT; DNA; ECG; EEG; EMG; GABA; HIV; MRI; NMDA; PET; PCR; RNA.
