# Supplementary material for: Moving the field forward: detection of epileptiform abnormalities on scalp electroencephalography using deep learning—clinical application perspectives
Source: Brain Commun. 2022 Aug 29;4(5):fcac218. doi: 10.1093/braincomms/fcac218 (PMC9453433; doi:10.1093/braincomms/fcac218)
Supplement: fcac218_Supplementary_Data [file Response_to_reviewers_comments_original.pdf › Response_to_reviewers_comments_original.pdf]

Reviewer: 1

This article aims to review the applications of deep learning to automate the detection of "epileptiform abnormalities". However, the main focus of the work is on the type of data and challenges the field of automated epileptiform abnormality detection faces, rather than on the methods that have been applied so far and the results they obtained.

A clear flow of information is lacking, and the goal of the review appears to change throughout the work (that is too lengthy, as well). Further, most illustrations must be improved, as well, and sometimes contain trivial details, too ("an EEG amplifier"). Detailed comments below (but are non-exhaustive)

Reply 1

We thank the reviewer for taking the time for reviewing this article and adopting a critical approach. We have taken the criticism into account and believe this has improved the article with the feedback. We have introduced a section on current DL studies and attached a supplementary file to reference the studies employed. We have also modified the illustrations based on the feedback for more clarity. The flow employed is of a discussion format with 9 main subheadings. We hope the replies below and the edited paper will reemphasize the aims and objectives of the paper. The paper does not intend to provide conclusive recommendations for all uncertainties in clinical aspects of deep learning but sheds light on issues which may be of importance in the success of DL beyond algorithmic technicalities and makes suggestions towards standardization and improved reporting.

MAJOR COMMENTS

-In the first paragraph of the introduction, the authors correctly distinguish machine learning (ML) form deep learning (DL). However, in the following paragraphs and throughout the article, ML is several times used to refer to DL approaches, suggesting it is a synonym

-The name of the first section, 'Overview of scalp EEG datasets available for machine learning' is once again inconsistent with the supposed DL focus of this work.

Reply 2: Thank you for this point. The articles which provided the observations for this narrative review were deep learning papers (now outlined in supplementary file 1). The heterogeneity we noted in reporting of metrics, data preparation, epileptiform labelling,

deficiency of external testing could be as well applied to machine learning studies given common paths in data preparation, IED verification, and performance testing. We have now minimized the term ML to avoid confusion. Where the term ML has been used, this will be in reference to questions or comments which are applicable to both e.g., benefits of automated detection, external validation, imbalanced dataset challenges, ethical implications and metric applications.

-In the introduction, one article employing DL methods is mentioned in detail without a convincing explanation of why it was chosen in detriment of other articles.

Reply 3**:** The study cited in the introduction made use of the largest dataset for a deep learning paper and appeared in one of the leading journals in clinical neurology. The introduction serves to present a view that although impressive results have been reported in literature the past few years, we are yet to see widespread automated IED detection implementation, open-source software available for download or even source codes/model details available on repositories for cross testing in other centres and to assess generalizability. There appears to be a gap between research output and clinical translation.

-The structure of the article is not logical: it goes from EEG data to performance testing, back to data annotation and then metrics evaluation, which is in turned followed by 'How should an IED model fit for clinical integration be reported – focus on the metrics' and then 'Whole EEG Classification'. There is no cohesive storyline throughout the article.

Reply 4**:** Thank you for this advice. We have made amends to the flow of the article and headings given its length to improve readability and restructured some sections of the article based on feedback given.

-In line 60 of page 6 / line 3 of page 7, one can read 'Amongst 101 subjects whose previous raw EEGs were accessible, 30% were noted to be misinterpreted as

epileptiform abnormalities.'. It is not clear what 'misinterpreted as epileptiform abnormalities' means.

Reply 5: This refer to benign-variant patterns being misreported as epileptiform abnormalities due to misinterpretation of EEG. We speculate that an automated algorithm may bias a non-expert EEG reader's judgement and report on benign variants as being epileptiform.

-The sentences in lines 3-10 of page 8 are unclear, and referring to a type of data as 'a common dataset' is scientifically incorrect.

Reply 6: We have changed this phrase to data type.

-The sentences in lines 50-58 of page 10 have no connection to the following paragraph, so it is not clear why they are placed in the beginning of that section.

Reply 7: We have edited the first few sentences for a better read. The purpose of the section is to introduce the clinical variety of EEG background and epileptiform abnormalities which exist in clinical epilepsy. Test data which is incongruent with training data may provide suboptimal models. A model trained on awake epileptiform abnormalities may present poor performance on EEGs which also contain various stages of sleep EEG and a range of artefacts in sleep. A dataset which has predominantly classical 3 Hz spike/wave won't generalise well to one which has a variety of polyspikes, diverse morphological focal sharps, paroxysmal fast or other atypical spike/wave morphologies.

-Figure 2 is composed of generalized EEG epochs, artefacts and other sub-images. The purpose of this figure is not clear, or why generalized epilepsy was chosen to represent pattern variability.

Reply 8: Genetic generalized epilepsy is a common type of epilepsy for which we have experimented several models in our centre. The figure serves to display a sample of the epileptiform variability which can exist within a single epilepsy type without exhausting the variation which can occur. We have now also added generalized paroxysmal fast (GPFA) and focal sharps to further demonstrate the diversity of IEDs and removed the amplifier

picture as recommended. The reason for the section as explained above is to give clinical insight into epileptiform abnormalities and the need for diverse labelling given the focus on ‘spike’ or ‘sharp’ model of detection targets displayed in publications. Each subfigure of an IED is different in its characteristics and quantitative analysis may show differences in various sample features deep learning models may learn from. This may include time-domain features like amplitude, duration, gradient of ascent or descent, entropy or data features in the frequency domain of the IED segment. Further these IEDs are more prolonged compared to spike target and sensitivity calculations can differ depending on discharge level on spike level analysis. For clinicians discharge level metrics are more informative.

-Figure 3 is composed of random EEG epochs that were supposedly misdetected by DL algorithms. However, it is not said by which algorithm, or how it was trained or with what type of data. Furthermore, it is only referenced once in the main text, between brackets, without any further explanation of its relevance.

Reply 9: This has now been rectified and we have edited the caption. We have now referenced figure 2 and 3 now in relevant paragraphs.

-The first sentence of section ‘Proposed Hierarchy for gold standard epileptiform detection’ is poorly written and the concept of ‘hierarchical order’ in this context is not explained to a satisfactory extent.

Reply 10: We have edited the initial sentence and the paragraph. We explain three levels in which IED data is labelled to determine ground-truth. One is using a more comprehensive review of patient’s ictal data (location, pattern), clinical background (radiology or other) and thus clinical judgement to validate data concordant sharp waves as IEDs. The second was based on the recent publication of the IFCN criteria as being objectively valid when compared to the first method with a focus on systematic criteria applied to waveform morphology. And the third was based on expert EEGers using experience with an adequate number agreeing on the sharp or spike abnormality being epileptiform.

-In the last paragraph of the introduction, this work is once more described as a ‘review’ that ‘summarizes some of the perspectives of clinicians’. It is not clear to which extent that is actually a review.

-Despite barely mentioning specific articles that use deep learning methods throughout this work, the authors refer to their own ‘ongoing work evaluating an unpublished dataset’ to support an argument. This does not make sense in such a review paper.

- Authors suggest this to be a review, and they should report to which extent the various performance metrics have actually been reported in the literature. There is not even an overview of the papers studied, what epilepsy types were included, if an independent set for evaluation was used, the size and type of the EEGs used, etc.

Reply 11: Many of the DL studies upon which the observations in this article are referenced in the manuscript. These include amongst others.

(Jing et al. 2019), (da Silva Lourenço, Tjepkema-Cloostermans, and van Putten 2021) (Wei et al. 2021), (Nhu et al. 2021), (Lourenço et al. 2020), (Fukumori 2019), (Prasanth et al. 2020), (Fürbass et al. 2020), (Xu et al. 2021)

We have now put the articles above plus others in a table format (See Supplementary Table) to give a more direct reference of the DL studies on which this viewpoint review was derived from.

The articles’ original goal was to complement a systematic review (currently under revision for *Journal of Neural Engineering*) which provided a technical and descriptive breakdown of recent deep learning studies in IED detection. The article is not intended to be an exhaustive coverage or a systematic review of deep learning methods and techniques. This paper covers an understanding on some of the clinical aspects such as nature of IEDs, EEG data types available and their possible implications, varied approaches to determine confidence levels of IEDs, metrics analysis and which performance measures translate into real world clinical use. We hope this will lead to improve understanding towards a clinical goal for deep

learning publications. This paper was not intended to critique articles one-by-one but provide a general sense of pitfalls which can occur in the field. For example, a paper may have a strength in focusing on clinically relevant metrics but may not provide details of data type, annotation methods or may not have an adequate gold standard used in identifying IEDs. Such clinical shortcomings make it difficult for another researcher to determine how the model would have worked on their private dataset.

We believe the foundation of optimal DL success in automated IED detection is dependent, amongst many factors (not all of which are within our insights), accurately labelled data with clinical awareness of epileptiform signal data and normal background as well as carrying out performance testing of models in a rigorous, transparent and methodologically sound manner. These factors have been the focus of this review.

Text oftentimes contains trivialities, including textbook knowledge on sensitivity/specificity/precision and more.. one may assume this is known by the readers of this journal.

- Metrics evaluation (page 18): this part is not clear, and authors should better distinguish between the performance of a particular algorithm and the relation to the priors. For instance, accuracy is a function of both the 'test quality' (i.e. the senstivity and specificity) AND the prior likelihood (with one exceptation in the unlikely circumstance that sensitivity and specifiticy are equal). Accuracy is therefore NOT the most intuitive measure to 'understand' (line 24) if different algorithms are compared (as typically priors are different).

Reply 12: Thank you for this input. We have reworded this sentence to avoid confusion as we are concordant with the argument that accuracy in isolation (mathematically defined as ratio of correct predictions to total predictions) or even AUC for that matter is not always interpolated to clinical accuracy. Prior likelihood may improve classification in a similar EEG test dataset as the training dataset from which a prior probability can be obtained or for which prior quantitative data is well established in literature. However, a wide range of factors may influence IED frequency burden in a random and diverse real-world EEGs of inpatient and outpatient epilepsy patients. This includes timing and setting of an EEG recording, epilepsy control at the time, anti-seizure medications, type and syndrome of

epilepsy, aetiology, sleep capture, source and depth of epileptogenic focus, application of various provoking stimuli amongst other clinical factors. This makes a generic prior probability for a universal detector challenging for a randomly presented test EEG.

On evaluation metrics

Reply 13: Although evaluation metrics are supposed to be standard textbook knowledge for medical doctors and basic researchers their implementation and terminology can differ. Recall and precision for example are not termed used widely in the clinical literature and their parallels sensitivity and PPV are used instead. Metric calculations can differ whether applied on a discharge or spike level (based on overlap with window) giving different results depending on which perspective is looked at. Other variation can be seen in literature. Specificity usually makes used of absence of abnormality in duration of segmentation used however in a few studies longer epochs may be used. In the Furbass study where specificity was based on true absence of IEDs in 11 and 14 second epochs which may or may not contained epileptiform abnormalities (median 1-8). The specificity was lower than all other papers reviewed because of the definition used rather than suboptimal model. One DL article in our review provided specificity values inconsistent with the usual definition throughout the article when they actually meant 1-specificity. Given a few of these inconsistencies it will be important for the author to be clear in the definitions they provide, and we have provided the usual definitions.

Minor comments
-In the abstract, the word ‘discharges’ is missing in the expression ‘inter-ictal epileptiform discharges (IEDs)’.
-The citation style is not uniform (eg. lines 41/42 of page 13).

Reply 14: Thank you for identifying this. This has now been corrected

Lines 56-60 of page 14,

Reply 15: We have retracted this sentence given an oversight as the statement was based on two studies. Details on IED method verification using video EEG had actually been elaborated by the authors in the discussion.

lines 8-13 of page 15 and

Reply 16: The references where IRAs have been moderate to high are cited in the next sentence in 46-48.

lines 52-54 of page 16 need citations.

Reply 17: The paper is now cited here.

Reviewer: 2

Comments to the Author
In this review Janmohamed et al review the existing approaches to automated interictal epileptiform abnormality detection, in particular the advent of machine (+deep) learning. They discuss the rationale, limitations, and evaluation of ML approaches. This is a broad and thoughtful piece that will inform the broad neurological community of such technology. The demand raised by the authors for open access to source code is welcome. Some minor points to consider are

1. Less is mentioned about how ML deals with the data (e.g. multivariate pattern analysis, independent component analysis, estimators, predictors) and more on the statistical output (such as AUC, specificity). So some information on common ML approaches might be helpful.

Reply 18: The supplementary excel table now supplied provides abbreviated details of the studies and some of the methods employed. We have also made mention of common automated detection methods used by researchers in the introduction briefly. The paper is being supplemented by a systematic review paper currently under review for details on technical aspects of deep learning studies. This paper did not focus on technical methods of machine and deep learning but on clinical aspects for improved modelling.

2. A table of the biggest ML studies in IED detection may be a good point of reference for readers

Reply 19: Thanks for this suggestion. We have now provided a table of the deep learning studies used in the preparation of this paper and their details.

3. One of the interesting future applications of such technology might be within the wearable realm and real time analysis of EEG data for the patient in home studies

Reply 20: Thank you for this suggestion. Seizure prediction devices may make use of spike detection algorithms and we have made a reference for this now.

4. Although consideration of montage of the data is mentioned, it would be useful to know if a particular montage is adopted for ML in the existing literature

Reply 21: We have now mentioned the montages most commonly applied in supplementary table.

5. Within the metric section one promising field that might be mentioned is a Bayesian approach to analysis (https://www.infona.pl/resource/bwmeta1.element.elsevier-b7bd05ba-b5ab-33bc-9054-086ec7163c6f)

Reply 22: Please see reply 12 to reviewer 1 in regard to prior probabilities being employed for classifiers.

6. Small point but when mentioning single channel evaluation (p21) there is the limitation of not appreciating the field associated with an IED, that would require neighbouring channel data

Reply 23: We have made a comment on this important point.

7. Also when mentioning time windows in p22 a small description of the difference between contiguous, sliding windows might be helpful

Reply 24: We have made mention of windowing in page 20 now.

8. Although likely outside the scope of this review, would be a ML approach to pathogenic high frequency oscillations

Reply 25: We thank the reviewer for the comment and agree that a discussion on this will be out of scope of the paper. The topic of HFOs using scalp recordings has potential prognostic utility in certain epilepsy conditions though is still in its infancy compared to automated iEEG HFO detection and application for localizing the epileptogenic zone. Several methodological obstacles yet exist in automated scalp HFO analysis which are yet t
