# Supplementary material for: Moving the field forward: detection of epileptiform abnormalities on scalp electroencephalography using deep learning—clinical application perspectives
Source: Brain Commun. 2022 Aug 29;4(5):fcac218. doi: 10.1093/braincomms/fcac218 (PMC9453433; doi:10.1093/braincomms/fcac218)
Supplement: fcac218_Supplementary_Data [file Revision_1_decision_letter.pdf › Revision_1_decision_letter.pdf]

From: brcoms.editorialoffice@oup.com

To: mubeen.janmohamed@monash.edu

CC:

BCC:

Subject: Brain Communications - Decision on Manuscript ID BRAINCOM-2021-396.R1

Body: 04-Jul-2022

Dear Dr. Janmohamed,

It is a pleasure to accept your manuscript entitled "Moving the field forward: detection of epileptiform abnormalities on scalp electroencephalography using deep learning.
Clinical application perspectives" for publication in Brain Communications. Any comments made by the reviewer(s) are included below.

The next step is for your paper to go through "First Look". We will contact you again shortly if any final files or formatting changes are required for publication or if we have any queries.

If no changes are needed, your submission will be transferred to our Production Department and you will hear from the Production Editor in due course.

VIDEO ABSTRACTS - Brain Communications encourages authors to provide a video abstract to accompany their article. These are hosted on the Brain/Brain Communications YouTube channel. If you are interested in producing a 5-min video abstract to promote your article, then please contact the Editorial Office (brcoms.editorialoffice@oup.com) for further details and guidelines.

PRESS RELEASES: If you are interested in coordinating the timing of any press releases or publicity with the publication of your article it may be useful to agree an embargo date. Please let us know in the Editorial Office (brcoms.editorialoffice@oup.com) as soon as possible so that we can start to put arrangements in place before we send your article to the Production Team.

Thank you very much for your contribution. On behalf of the Editors of Brain Communications, we look forward to your future contributions to the Journal.

Best wishes

Prof. Richard Chin
Associate Editor, Brain Communications

Professor Tara Spires-Jones
Founding Editor, Brain Communications

Reviewer(s)' Comments to Author:

Reviewer: 2 Umesh Vivekananda

Comments to the Author
Thank you for addressing my points. I think it would be helpful for the reader to know that this paper supplements a more technical one that has been submitted, to give a more comprehensive overview.
